# Internet of Things Technologies and Machine Learning Methods for Parkinson’s Disease Diagnosis, Monitoring and Management: A Systematic Review

**DOI:** 10.3390/s22051799

**Published:** 2022-02-24

**Authors:** Konstantina-Maria Giannakopoulou, Ioanna Roussaki, Konstantinos Demestichas

**Affiliations:** 1 School of Electrical and Computer Engineering, National Technical University of Athens, 15773 Athens, Greece; kgiannakopoulou@cn.ntua.gr (K.-M.G.); cdemest@cn.ntua.gr (K.D.); 2 Institute of Communication and Computer Systems, 10682 Athens, Greece

**Keywords:** Parkinson’s disease, wearable technology, sensors, internet of things, artificial intelligence, machine learning, deep learning, remote monitoring, smart personalized healthcare

## Abstract

Parkinson’s disease is a chronic neurodegenerative disease that affects a large portion of the population, especially the elderly. It manifests with motor, cognitive and other types of symptoms, decreasing significantly the patients’ quality of life. The recent advances in the Internet of Things and Artificial Intelligence fields, including the subdomains of machine learning and deep learning, can support Parkinson’s disease patients, their caregivers and clinicians at every stage of the disease, maximizing the treatment effectiveness and minimizing the respective healthcare costs at the same time. In this review, the considered studies propose machine learning models, trained on data acquired via smart devices, wearable or non-wearable sensors and other Internet of Things technologies, to provide predictions or estimations regarding Parkinson’s disease aspects. Seven hundred and seventy studies have been retrieved from three dominant academic literature databases. Finally, one hundred and twelve of them have been selected in a systematic way and have been considered in the state-of-the-art systematic review presented in this paper. These studies propose various methods, applied on various sensory data to address different Parkinson’s disease-related problems. The most widely deployed sensors, the most commonly addressed problems and the best performing algorithms are highlighted. Finally, some challenges are summarized along with some future considerations and opportunities that arise.

## 1. Introduction

### 1.1. Parkinson’s Disease

Parkinson’s disease (PD) is the second most common neurodegenerative disease [1] and is responsible for a considerable amount of disability-adjusted life years and deaths globally [2], leading to an extremely high demand for respective health resources. In recent decades, we have witnessed a dramatic rise in the amount of people suffering from it worldwide, which is correlated with the ageing of the global population, as well as with other potential factors, such as air pollution and smoking [1,2]. PD is related to the prominent loss of dopaminergic neurons, the development of Lewy bodies and neuroinflammation [3]. It is attributed to a complex combination of genetic and environmental factors [1,3]. However, the exact cause of PD remains unknown.

PD is manifested with mainly motor symptoms, such as resting tremor, muscular rigidity, bradykinesia, or even akinesia, postural and gait impairment, but is also related to non-motor characteristics, such as sleep dysfunction, autonomic dysfunction, including orthostatic and postprandial hypotension, fatigue, pain, hyposmia, bladder and gastrointestinal disturbances, cognitive deficits, depression, mood disorders, dementia and hallucinations [1,3,4,5]. There are also indications that phonation and speech disorders are common early signs among PD patients [6,7]. These clinical symptoms may be manifested differently from patient to patient, as some of them may be absent, while others may be quite severe. Similarly, the progression of the disease varies across PD patients. This phenotypic variability has led to the definition of several PD subtypes. One of the most widely accepted classifications corresponds to tremor-dominant PD and non-tremor-dominant PD [8]. Additionally, the manifestations of the disease demonstrate fluctuations in the same patient, in concordance with the ON and OFF states, which are also related to the impact of the levodopa treatment [9]. This is one widely proposed treatment for the management and alleviation of PD symptoms, without however curing the disease.

Νon-motor symptoms may be present earlier than the motor ones. However, clinical diagnosis is performed based on the latter, including the presence of bradykinesia, tremor or rigidity [5], as the former are often not easily detected and are usually attributed to other health factors and to senescence. Furthermore, there is no specific test for PD diagnosis. Conventionally, the patient’s medical history, signs, symptoms and medical examinations are taken into consideration by the doctor, including some simple physical examinations and exercises and some mental tasks, to finally diagnose PD. Brain images, such as magnetic resonance images (MRIs), computed tomography (CT) scans, positron emission tomography (PET) scans and radiographs, or other lab tests may also be exploited to exclude other medical conditions [10].

The assessment of the PD symptoms is usually accompanied by some scores in common PD-related motor or non-motor rating scales, such as the Movement Disorders Society Sponsored Revision of the Unified Parkinson’s Disease Rating Scale (MDS-UPDRS), the Hoehn and Yahr (H&Y) Staging Scale, the Schwab and England Activities of Daily Living (ADL) Scale, as well as some self-administered questionnaires, such as the Self-Assessment Parkinson’s Disease Disabilities Scale (SPDDS) [11]. The aforementioned clinical tests and the respective rating scales are also used to estimate the progression of the disease during regular follow-up appointments with the neurologist.

The lack of well-established PD biomarkers and the similarity of manifestations among different neurological disorders complicate and delay successful diagnosis. Furthermore, the patient may suffer from several fluctuations between two consecutive doctor’s appointments, which makes diagnosis and monitoring based on clinical examinations more difficult, especially when patients fail to precisely report their symptoms. Consequently, the patient’s clinical picture is subsampled, and useful information may vanish. Additionally, both inter-subject and intra-subject variability concerning PD clinical symptoms highlight the urgent need for personalized treatment suggestions and complicate the relevant medical decision-making processes. Provided that the diagnosis is performed early enough and the clinicians have adequate available information when monitoring a PD patient, the impact of the tailor-made treatments is expected to be greater.

### 1.2. Atrificial Intelligence and Internet of Things for Parkinson’s Disease Diagnosis and Management

Artificial intelligence (AI) is the field of computer science which deals with intelligence attributed to machines. According to Russel and Norvig [12], intelligent agents perceive their environment and make decisions in order to maximize their chance to achieve their goals. Conventionally, AI is rule-based and makes use of human experts’ knowledge. Machine learning (ML) is a more flexible and data-driven subfield of AI, according to which a computational machine may improve its performance in a specific task through acquiring experience [13], imitating the way that humans learn. Furthermore, deep learning (DL) is a subfield of ML which is less dependent on human intervention and is based on artificial neural networks (ANNs) that consist of multiple neurons stacked in many different layers and connected with each other, inspired by the structure of the human brain [14]. ML and DL algorithms and techniques can be applied to data collected from sensors, embedded on wearable devices or other everyday objects. Nowadays, these data are abundant and easy to acquire thanks to the Internet of things (IoT) paradigm, which enables various physical objects with embedded sensors and unique identifiers to communicate with each other and transfer data over a network [15].

AΙ and ΙοΤ technologies can contribute to both the early diagnosis of PD and PD patients’ monitoring, aiming to enable improved personalized treatments and assess the proposed ones [16], complementing the already established conventional methods. Moreover, by training ML algorithms on sensory data collected from PD patients, evaluation of suitability of current prescribed medication can be facilitated, as well as the optimization of surgical treatments, the prediction of the course of the disease and the prevention of undesirable consequences even in real time [16]. These interventions pave the way for precision medicine in PD, among other chronic diseases, and support transition from clinic-centric to patient-centric healthcare approaches. Finally, the opportunities that arise from remote monitoring with the help of wireless sensors, other wearables and telemedicine tools may cover the respective urgent need in the COVID-19 pandemic era and help PD patients to continue their care and treatment without putting themselves at risk [17], overcoming at the same time the spatial and temporal constraints in place.

More specifically, ML and DL techniques have been applied to brain images and other conventional clinical examinations to implement computer-aided diagnosis (CAD) tools [18]. These approaches are of utmost importance, as they may extract features that are not easily detectable by experts. However, the respective published studies will not be discussed in the current literature review, which is dedicated to IoT interventions combined with AI methods. Sensors are a valuable source of information that can feed ML and DL algorithms with rich data, collected during or between doctor’s appointments, in laboratories, hospitals or in free-living environments, at a reasonable cost. All these wearable or non-wearable sensors are networked and can transmit data from mobiles, tablets or other smart devices to remote databases by exploiting the IoT infrastructure available [19].

The vast majority of the deployed sensors include inertial sensors or inertial measurement units (IMUs), such as accelerometers, gyroscopes and magnetometers [20], as well as pressure sensors and ground reaction force (GRF) plates measuring features correspondent to PD motor symptoms [21,22]. Moreover, according to Monje et al. [21], there are several other technologies deployed at various technology readiness levels. For example, firstly, image and depth sensors, as well as sound or audio sensors have been also used to capture motor and non-motor PD manifestations. Other deployed sensors that capture various bio-signals include electroencephalography (EEG), electrocardiography (ECG) and electromyography (EMG) sensors, pain measurement devices, portable sleep measurement devices and polysomnography (PSG) sensors, eye tracking systems, heart rate and temperature sensors, among others. Finally, these data sources and the respective ML and DL models trained are sometimes integrated in mobile applications [23,24], conversational agents or chatbots [25] and serious games [26] and can be combined with controllers [27] to improve the patient’s quality of life.

### 1.3. Aim of the Current Systematic Review

The purpose of the review presented in this paper is to investigate the use of ML and DL models trained on sensory data, in support of PD patients, their caregivers and the clinicians in every phase of the disease. Therefore, it summarizes the main findings of various research articles that propose PD predictive and estimation models based on novel IoT technologies and optimal sensor deployments. The aim is to shed light on more novel approaches and present new possibilities that have not been discussed to a sufficient extent in the previously published reviews, providing neurologists with useful insights that could potentially revolutionize PD diagnosis and care. For example, there are some review studies in the current literature that summarize different types of sensors and commercially available devices, which are deployed to monitor PD patients with respect to various aspects and motor symptoms, without however emphasizing predictive ML models [20,21,22,28,29,30,31]. There are also others that focus only on one problem, such as rigidity quantification [32], impaired gait analysis [33,34,35], freezing of gait episodes and potential falls detection [36,37,38], as well as computer-aided PD diagnosis [39,40]. The current systematic review intends to fill all these gaps, by presenting a more holistic approach, examining both the sensorial and the algorithmic side of strictly IoT- and ML-based approaches.

Nevertheless, there are some other preceding reviews that address this topic, following a similar approach [41,42,43,44,45,46,47]. However, the approach adopted herein is more comprehensive than most of these systematic reviews, as 112 studies are analyzed and considered, compared to 10–48 studies considered in previous approaches. Only Rovini et al. [41] examines 136 papers, but this systematic review was published in 2017 so naturally does not consider all approaches published in the last four years. Moreover, the review presented in this paper sheds light on additional questions, such as which the most commonly addressed problems regarding PD are, which the most deployed sensors are, and which the most used and best-performing ML algorithms are. Finally, the review of this paper highlights not only the added value of these interventions, but also the challenges and the potential pitfalls that they involve, as well as some gaps and open issues, proposing some future directions for improvement.

## 2. Methods

The current literature review is conducted in a systematic way that is largely consistent with the latest PRISMA guidelines [48]. As already stated, the purpose of this review is to summarize the advances in AI approaches based on data collected through sensors and IoT technologies that support PD patients and neurologists in every phase of the disease. On this basis, the authors made the following decisions regarding the identification of search strategy and data sources, the criteria for the final selection of the studies and the selection and data extraction processes.

### 2.1. Literature Search Strategy

Firstly, the most suitable databases and registers of the respective literature were selected. The authors opted for three popular academic citation databases, which guarantee the high quality and impact of the considered papers. The first one is PubMed, one of the most popular databases of biomedical literature. The second one is IEEE Xplore, which is a well-known database of technical literature and the third one is Scopus, which indexes academic articles from a wide range of research fields.

Then, the most suitable keywords were selected, to assure the inclusion of studies that would answer the posed research questions. Indicative such terms are “Parkinson’s disease”, “artificial intelligence”, “machine learning”, “deep learning”, “sensor”, “internet of tings” and some specific types of sensors, such as “accelerometer”, “gyroscope”, etc. All these terms were combined with logical expressions. Additionally, some other keywords were added, such as “remote”, “portable”, etc., to ensure that clinical equipment which cannot be deployed for remote diagnosis or monitoring would be excluded. The selection of the final technical keywords was based on methods and devices discussed in previous review articles, after several adjustments to ensure the inclusion of all the technologies of our interest. Finally, the considered studies should be articles published in journals or in conference proceedings in the last decade (from January of 2012 till August of 2021) and written in English. During the last decade, the exploitation of AI algorithms and IoT tools in healthcare was hyped. This period is also long enough to support a comprehensive review approach with concrete conclusions about the evolution of the respective methods proposed regarding PD over the years.

After this fine-tuning, the final search query that was submitted to the Scopus database is the following: TITLE-ABS-KEY (“Parkinson’s disease” AND (“machine learning” OR “deep learning” OR “artificial intelligence”) AND (sensor OR device OR “internet of things” OR “accelerometer” OR “gyroscope” OR imu OR “inertial measurement unit” OR “force platform” OR “force plate” OR video OR camera OR smartphone OR smartwatch OR ((electromyography OR electroencephalography OR polysomnography OR electrocardiography) AND (portable OR wearable OR home OR remote)))) AND PUBYEAR > 2011 AND LANGUAGE (English) AND DOCTYPE (ar OR cp). Equivalent, semantically and syntactically, queries were submitted to the other two databases as well.

### 2.2. Eligibility Criteria

Some inclusion and exclusion criteria were defined to ensure that the research questions are answered. These eligibility criteria can be summarized as follows:

- Firstly, the considered studies should be strictly articles or conference papers, published between January 2012 and August 2021, as already stated.
- The considered studies should be about any aspect or phase of PD. The hypotheses should be tested on adult human subjects, under strict ethical guidelines. Some of the subjects should be PD patients but healthy subjects and other neurological disorders patients may also be included for, e.g., differential diagnosis. However, articles that present technologies which can be leveraged for PD diagnosis or management but are tested only on mixed or healthy populations are excluded. Furthermore, there should be a concise description of the datasets used and the respective signals should be real and not simulated.
- Moreover, the signals used should derive directly from wearable or non-wearable sensors, smart devices, ambient or other technologies that are related to IoT. Studies that exploit data from other sources, such as conventional medical equipment, interviews or medical reports that cannot be adopted for remote monitoring or diagnosis are excluded.
- At the same time, at least one specific AI algorithm should be proposed to solve the corresponding PD-related problem. The respective ML models should be trained over datasets that are in accordance with the previous guidelines and their performance should be measured with specific evaluation metrics. Studies that present only a statistical analysis and not any AI methods are excluded. Finally, the provided conclusion should be consistent with the initial research goal.

In addition to the previous guidelines and restrictions, since multiple studies may repeatedly address the same problems, proposing the same ML methods based on the same input data, some studies were clustered in groups and only some discrete representatives of each group were considered to avoid redundancy. More specifically:

- If there are both a conference paper and an extended research article for the same study, the latter is preferred.
- In the case that the same or similar PD-related problems are addressed by one research group multiple times, utilizing similar methods or same datasets (e.g., proposing one or two more algorithms each time), only one is preserved and the rest are excluded. The most recent or comprehensive and detailed one was preferred.
- Finally, even if the research group is different when there are two or more almost identical approaches, only one is presented and the rest are excluded. By the term identical approaches, the paper refers to the exact same PD-related task, same ML/DL algorithms and the exact same open dataset or similar train and test population.

### 2.3. Selection and Data Collection Processes

One investigator (K.M.G.) submitted the search queries to the three selected databases. The studies were retrieved automatically with the help of Zotero (https://www.zotero.org/, accessed on 17 February 2022). The same investigator proceeded to the removal of duplicate records, again automatically by the Zotero tool. Then, the investigator (K.M.G.) screened the title and the abstract of the remaining studies to remove further articles, according to the inclusion and exclusion criteria set, without clustering similar approaches at this initial stage. Moreover, the investigator (K.M.G.) identified and excluded a few studies, for which the full text could not be retrieved. At this point, two investigators (K.M.G. and I.R.) screened independently the full text of the remaining studies. Each investigator concluded independently to a set of studies to be considered in the current literature review, according to the inclusion and exclusion criteria set. Then, the two investigators (K.M.G. and I.R.) discussed their conclusions. The agreement rate was high and after solving all the divergences, they reached a consensus about a final set of studies to be considered. The two investigators also agreed upon the information that should be collected from each report and the first evaluator (K.M.G.) finally performed the data collection process from each study individually, according to what has been discussed.

## 3. Results

### 3.1. Search Results

**Figure 1:**
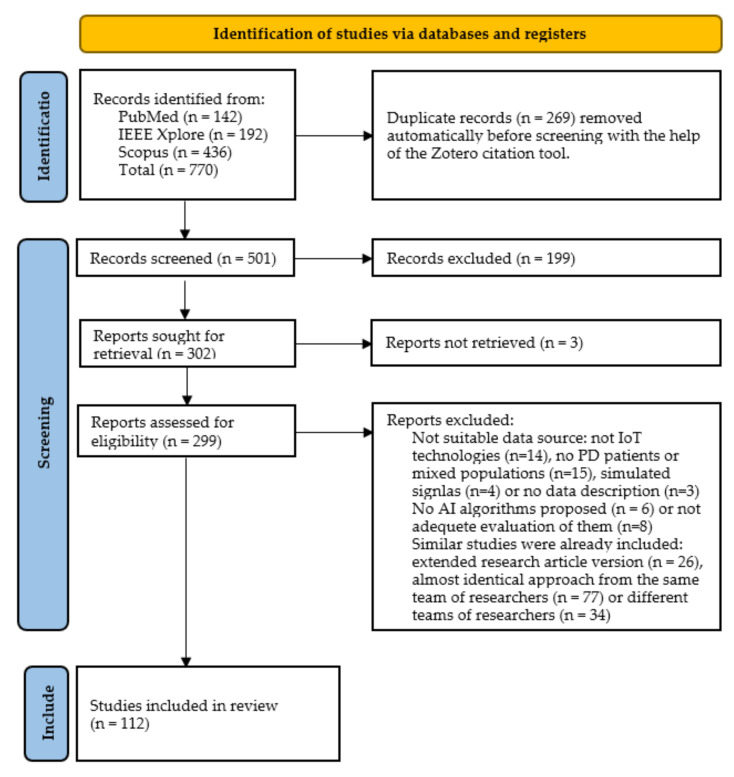
Flow diagram of the PRISMA methodology followed in the literature review.

The steps of the PRISMA-based methodology discussed in Section 2, along with the results obtained at the end of each phase are illustrated in the flow diagram depicted in Figure 1. Initially, 770 articles were retrieved, after submitting the queries to the three databases (142 articles from PubMed, 192 articles from IEEE Xplore and 436 articles from Scopus). Next, 269 duplicates were removed, resulting in 501 studies to consider. Then, other 199 reports were excluded, after screening the title and the abstract of the remaining studies, according to the inclusion and exclusion criteria. Moreover, the full text of three studies could not be retrieved via the web, resulting in 299 reports to be assessed for eligibility. Eventually, 112 studies were selected to be considered in the literature review presented in this paper, after screening the full text of each study according to the inclusion and exclusion criteria set.

**Figure 2:**
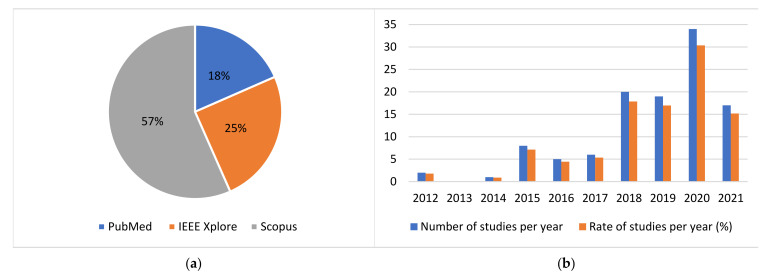
Distribution of (**a**) studies retrieved per academic citation database and (**b**) studies considered per year.

The distribution of the initially retrieved studies per academic citation database is depicted in the pie chart of Figure 2. The vast majority of the studies which were finally selected to be analyzed in the current review, are listed in more than one of the considered databases. Thus, the respective pie chart for the finally considered studies is not presented, as it provides no significant additional information.

Moreover, it is substantiated that the results obtained are up to date. More specifically, the distribution of the publication years of the considered papers is depicted in the column chart of Figure 2. As one can easily observe, less than 20% of the considered studies are published before 2018, while the rest are published in the last 4 years. It is worth mentioning that more than 30% of the studies considered are published in 2020. Additionally, the respective rate for 2021 is also expected to be higher, as this literature research was conducted in the August of 2021 and the remaining 4 months of the year are not considered. To conclude, the results reached by the present study may serve as a complement of the preceding comprehensive systematic review conducted by Rovini et al. [41] in 2017, providing additional state-of-the-art information and insights from the last 4 years.

### 3.2. Results of Individual Studies and Sensor-Wise Synthesis

The selected articles are divided into six major categories according to the types of sensors and IoT technologies deployed. These technologies span across inertial sensors, pressure or force sensors, image and depth sensors, audio or voice sensors, other wearable biosensors or other types of sensors in general that are not considered in the previous categories and the combination of different types of the aforementioned sensors. For each category, the studies that address the same or similar PD-related problems are grouped and the main points of the respective approaches are briefly presented. Furthermore, the performance of the various AI algorithms deployed are compared with each other and an attempt is made to identify the methods that outperform the rest with respect to a specific PD-related task based on one specific type of sensory input data each time.

Finally, the key parts of each study discussed, including the input data, the population of the dataset, the problem addressed, the deployed algorithms and their performance on the basis of specific evaluation metrics, are also summarized in six comprehensive tables in Appendix A of this paper, which are again divided according to the six different categories of sensors introduced in the current section.

#### 3.2.1. Inertial Sensors

The vast majority of the considered articles are based on wearable inertial sensors or IMUs, such as accelerometers, gyroscopes, magnetometers and other angular velocity sensors, which measure several kinematic parameters. The studies considered in this category provide evidence that these measurements may shed light on various aspects of PD, mostly related to motor symptoms, supporting PD diagnosis, differential diagnosis between various neurological disorders, identification of PD different subtypes, detection of PD symptoms and estimation or prediction of their severity, detection or prediction of FoG episodes, detection of symptoms fluctuations, segmentation of gait, rehabilitation assessment, as well as estimation and prediction of patients’ response to the prescribed medication.

There are 52 studies in this category that make use of inertial sensors solely, out of which 21 studies correspond to lower-limb analysis, 21 studies refer to upper-limb analysis and 10 studies correspond to whole-body analysis. Firstly, acceleration and angular velocity signals collected with IMUs attached to the waist or to the feet at several positions can provide useful insights regarding impaired gait ability or postural instability due to PD. Similarly, acceleration and angular velocity signals from hand-mounted inertial sensors can shed light on PD-related tremor parameters, bradykinesia and dyskinesia symptoms. IMUs can also be attached to other body parts, such as the sternum to extract more motor-related features, which are possibly correlated with the course of PD. Finally, these sensors are sometimes embedded in smartphones, smartwatches or other everyday objects. More details about the respective review findings for this type of sensorial data exploited are provided in Table A1 in Appendix A of this paper.

The first problem that will be discussed is PD diagnosis or equivalently the classification between PD patients and healthy controls, which is frequently addressed based on inertial signals. To this end, gait parameters have been extracted either manually via feature engineering techniques [49,50,51,52,53] or automatically via deep convolutional neural networks (CNNs) [54], to feed several classification algorithms. The deployed algorithms include support vector machines (SVMs), decision trees (DTs), random (RFs), bagged, boosted and fine trees, k-nearest neighbors (kNN), logistic regression (LR), linear discriminant analysis (LDA) and naïve Bayes (NB) classifiers, as well as multi-layered perceptrons (MLPs) or other neural networks (NNs). It appears that when IMUs are attached to the feet, researchers achieve slightly higher performance than when sensors are attached to the waist or to the lower spine. For example, in [49,51,53], PD diagnosis is performed with 90–99.33% accuracy by a DT, an MLP and an RF classifier, respectively, trained on feet signals, while 84.5–85.51% accuracy is obtained when kNN algorithms are trained over waist signals [50,52]. Moreover, the performance does not seem to improve significantly when deep CNNs are deployed in [54], leading to 0.87 area under the receiver operating characteristic curve (AUROC).

Similarly, features extracted from upper-limb motor analysis can be leveraged for PD diagnosis. In concordance with the previous case, various ML classifiers, including NB, LR, kNN, SVM, adaptive boosting (AdaBoost), DT, RF, gaussian mixture model (GMM) and deep neural networks (DNN) classifiers, have been proposed in the literature to address this problem. To elaborate, tremor measurements have been obtained with the help of inertial sensors embedded in smartphones [55], Wii Remote [56] and spoon handles [57] to differentiate PD patients from healthy controls by exploiting conventional ML algorithms. RF and SVM models perform very well, achieving 0.94 and 0.98 AUROC, respectively. Similarly, signals from wrist-worn inertial sensors have been exploited for this cause. In [58], a moderate classification accuracy of 89% is achieved with the help of SVMs, while in [59], gait patterns are extracted from wrist-acceleration signals and detect PD patients with GMMs, achieving AUROC = 0.85. Finger-mounted sensors have also been used for recording motor data during finger-tapping or other hand movements for PD diagnosis. For example, Park et al. [60] exploit finger-mounted IMUs to train DNNs and achieve AUROC = 0.888–0.95. As one can easily observe, similar performance is obtained for all these approaches. In [59], the GMMs, which contain an unsupervised learning phase, perform slightly worse than the rest methods, while the DNN in [60] does not outperform significantly the conventional ML models.

It can be concluded that there is no significant difference between lower-limb and upper-limb analysis, regarding the performance of the proposed models for PD diagnosis based on IMUs. Moreover, it should be highlighted that sensor networks combining multiple inertial sensors’ signals from various body parts placements have been also deployed for PD detection. However, their exploitation has not significantly improved the classification performance of the previously discussed studies. Some indicative approaches are [61,62,63,64]. In [62], an SVM classifier achieves 95% accuracy, while in [63], an RF classifier achieves 86–94.6% accuracy. The highest performance (96% accuracy) is obtained in [64], with the help of a majority voting scheme over several conventional ML algorithms, while the lowest performance (68.64–73.81% accuracy) is obtained in [61] with an MLP, trained with features extracted by a convolutional autoencoder (AE), which was pre-trained on healthy subjects’ data.

Besides binary classification between PD patients and healthy people, differential diagnosis between several neurological disorders with similar manifestations can be achieved with the help of wearable inertial sensors and ML algorithms. For example, in [65,66], signals from hand-mounted inertial sensors are used to train ML models, such as RFs and SVMs, to detect PD among other neurological disorders and achieve a moderate accuracy of 72–79%. Moreover, in [67], PD patients are efficiently differentiated from essential tremor (ET) patients by an SVM trained on smartphone angular velocity signals, with 77.8% accuracy. A bit higher classification performance (89% accuracy) is achieved by Moon et al. [68] when addressing the same problem with NNs trained over fused signals from sternum-, lumbar-, wrist- and foot-worn inertial sensors. Similarly, De Vos et al. [69] managed to differentiate PD patients from progressive supranuclear palsy (PSP) patients by training an RF with signals from multiple IMUs placed at various body places, achieving 88% accuracy. Finally, gait features can classify different forms of parkinsonism with 73.33% accuracy, by deploying deep belief networks (DBNs), as indicated in [51]. As one can easily observe, SVMs, RFs and NNs are usually chosen as the best option to address differential diagnosis. Finally, the models detect PD among other diseases with significantly higher accuracy when multiple IMU signals are combined from various body placements, as seen in [68,69].

Inertial sensors have been also used for the quantification of PD-related symptoms and the estimation of the respective scores in well-known scales, such as the UPDRS and the H&Y scale. In [52], walking features are extracted from lower spine signals with a hidden Markov model (HMM) and train a kNN model to finally estimate 36-Item Short Form Survey (SF-36) scores with 27.81 mean absolute error (MAE). Similarly, gait features extracted from smartphone inertial signals can classify MDS-UPDRS severity levels with AUROC ranging between 0.92 and 0.97, by training ANNs and RF or bagged trees models [53,70]. It seems that the last two classification tasks are easier to be performed successfully than the regression one, presented in [52]. Nevertheless, it needs to be taken into account that the output scales are different in each case and that the used signals in [53,70] derive directly from the feet and not the lower spine.

Furthermore, signals from hand-mounted inertial sensors have been exploited to extract features either manually and train conventional ML models [71,72,73,74] or automatically and train DL models, such as CNNs [72], to estimate UPDRS scores. All these approaches exploit wrist acceleration or angular velocity signals, except for the study [73], where the authors make use of finger-mounted sensors. In [71], 91% accuracy is achieved with an MLP; in [72], 85% accuracy is achieved with a CNN; in [73], 96–97.33% accuracy is achieved with an RF; while in [74], 0.79–0.88 AUROC is achieved with an LR model. Unexpectedly, the DL approach [72] performs a bit worse than other conventional ML approaches, but the different placement of the sensors may have influenced the results. Finally, H&Y scale scores have also been estimated with SVM models based on tremor measurements from inertial sensors embedded in everyday objects, achieving accuracy up to 77% and correlation coefficient up to 0.97 [56,57]. Consequently, in the considered studies, PD patients are classified according to their H&Y scores with slightly lower accuracy than when they are classified according to their UPDRS scores.

Moreover, sensor networks that capture both upper-limb and lower-limb motion characteristics have been deployed for PD severity estimation as well. For example, in [75], a random undersampling boosting (RUSBoost) algorithm based on full-body inertial signals estimates H&Y scale scores, without however outperforming the previous approaches, reaching up to 78% accuracy and 0.87 AUROC. Nevertheless, in another approach [64], SVM models achieve 87.75–94.5% accuracy, which corresponds to the highest performance obtained for H&Y scale. Additionally, in [76,77] wrist- and foot-worn IMU signals are combined to quantify PD severity. In the first case, CNNs and long short-term memory (LSTM) networks are combined to estimate UPDRS scores, while in the second, the adaptive neuro-fuzzy inference system (ANFIS) is proposed. Correlation coefficient reaches up to 0.79 and 0.814, respectively. Similarly, failures of PD patients to follow the MDS-UPDRS III movement protocol have been identified with various ML algorithms and the respective accuracy reaches up to 78% [78]. In conclusion, it seems that the performance of PD severity estimation models is independent of the placement of the sensors, as upper-limbs, lower-limbs and combinational approaches demonstrate similar performance regarding this task.

Additionally, sometimes the researchers address the detection of PD-related symptoms and do not proceed to the quantification of their severity. This is the case in the studies that are presented below, where hand-mounted IMUs are used to train ML models to detect tremor and bradykinesia. Firstly, in [79] bradykinesia is detected in PD patients with 90.9% accuracy by training DNNs over hand accelerations. Similarly, Shawen et al. [80] detect both bradykinesia and tremor with the help of an RF classifier, achieving AUROC up to 0.77 and 0.79, respectively. Tremor detection, accompanied by the estimation of the respective duration, are also performed by training an MLP classifier with automatically extracted features from a CNN, obtaining AUROC = 0.887 [81]. The tremor onset can be identified with an MLP trained over manually extracted features with 92.9% accuracy, as well [82]. Finally, Channa et al. [83] discriminate PD patients with tremor from PD patients with bradykinesia and from healthy controls, with the help of a kNN model, achieving 91.7% accuracy. In conclusion, it is observed that the RF classifier in [80] performs poorer than the MLP, DNN and kNN models in the rest approaches.

On the other hand, lower-limb analysis with inertial sensors can support the detection of freezing of gait (FoG) episodes. In [84], accelerometer and gyroscope signals feed an AdaBoost classifier and detect FoG with sensitivity ranging between 81.7% and 86%. For the same cause, DNNs have been deployed, such as CNNs [85,86], LSTMs [87] or their combination [88]. The highest accuracy (91.9%) is obtained with the combination of a CNN and an attention-enhanced LSTM [88], while CNNs and LSTMs alone achieve 89% and 83.38% accuracy, respectively [85,86,87]. Furthermore, ML algorithms trained with inertial sensors signals may predict FoG episodes in a short time of period, or equivalently may classify gait sequence segments in walking, pre-FoG, FoG or post-FoG/walking phases. This problem is addressed in several studies [89,90,91,92,93,94] that mainly use the open Daphnet dataset (https://archive.ics.uci.edu/ml/datasets/Daphnet+Freezing+of+Gait, accessed on 17 February 2022). Many algorithms have been deployed to address this task, including LSTM, SVM, kNN, MLP, extreme gradient boosting (XGBoost), RF, gradient boosting machine (GBM), LR and LDA models among others. In [89], 85–95% accuracy is obtained with an LSTM NN; in [90,91,94], 77–86.1% accuracy is obtained with SVMs; in [93], 83% accuracy is obtained with LDA; while in [92], 98.92% is obtained with a kNN classifier. Generally, in this case, it can be concluded that DL approaches tend to perform better than the ones that make use of conventional ML models. Finally, gait features extracted from feet-worn IMUs can also be leveraged for strides detection or gait segmentation of PD patients with the help of hierarchical HMMs [95]. The respective f1-score ranges between 95.9% and 100%.

Besides PD symptoms monitoring, inertial sensors can be used for the monitoring of treatment-related fluctuations and the detection of treatment-induced adverse symptoms. In [96,97], on and off medication states are detected based on gyroscopic signals from an ankle-mounted sensor and acceleration signals from a knee-worn sensor, respectively. In the first case, an LSTM NN is deployed, achieving 73–77% accuracy, while in the second, an RF classifier is trained, and accuracy reaches up to 96.72%. In addition, in [98], a third class is added which corresponds to the levodopa-induced dyskinesia. A CNN is trained with acceleration signals collected from a wrist-worn sensor in a free-living environment and discriminates the three classes with 65.4% accuracy. The levodopa-induced dyskinesia can also be detected, as a binary classification problem, by training SVMs with wrist or hip acceleration and angular velocity signals, obtaining 65–82% accuracy [99]. In this case, as one can observe, DL approaches demonstrate moderate performance and do not outperform the conventional ML ones. As a matter of fact, higher classification accuracy is obtained with RF and SVM models than with CNN or LSTM networks.

Moreover, the response to the levodopa treatment can be estimated based on accelerometer or gyroscope data from wrist-worn sensors. In [74], a classification approach is presented which makes use of an LR model, with AUROC ranging between 0.82 and 0.92. On the other hand, in [58], a support vector regressor (SVR) estimates the exact values of the treatment response index with 0.69 root mean square error (RMSE). Finally, Watts et al. [100] first cluster with k-means PD patients according to their response to prescribed medication and then they classify them to these groups based on their motor characteristics. In this approach, 86.9% accuracy is obtained with an RF classifier. It is worth mentioning that none of the retrieved studies exploits DNNs to address the treatment response estimation problem.

#### 3.2.2. Pressure and Force Sensors

Another popular type of sensors for PD diagnosis and monitoring is the one that measures applied force or pressure. The results of the current literature research indicate that these sensors have been mainly used for PD diagnosis, differential diagnosis and PD motor symptoms quantification. A group of seven relative studies that train ML models over the signals produced by these sensors to address PD-related problems is presented in Table A2 in Appendix A of this paper. As one can observe, only lower-limb analyses are considered. Even though pressure sensors can be embedded in everyday objects for upper-limb motion measurements as well, to the best of the authors knowledge, there has been no study where these sensors have been used stand-alone to collect the necessary data. Therefore, the respective studies that combine such sensors with others for upper-limb observations are presented in Section 3.2.6, which is dedicated to the combinational analysis of multiple types of sensors.

In concordance with the inertial sensors studies, PD diagnosis and severity estimation are commonly addressed problems and are discussed in five out of the seven studies considered [101,102,103,104,105]. In all these approaches, an open dataset [106] that merges three gait datasets, with vertical GRF signals from eight in-sole sensors in each foot, is exploited. More specifically, PD diagnosis is performed by training a feed-forward DNN [102], a 1D-CNN [103] and a dual-modal CNN accompanied by an attention-enhanced LSTM [104], with 99.29–99.52%, 98.7% and 99.07–99.31% accuracy, respectively. In all these cases, DL approaches achieve almost excellent classification accuracy, outperforming other conventional ML algorithms tested.

At the same time, these studies estimate the severity of PD-affected gait, with respect to the H&Y scale, achieving 98.57–99.1% [102], 85.3% [103] and 98.03–99.01% accuracy [104], respectively. In this case, the feed-forward DNN and the dual-modal CNN with the attention-enhanced LSTM clearly outperform the CNN approach, maintaining again an exceptionally high accuracy, greater than 98%. PD patients have been also classified according to their H&Y scores with the help of a time delay NN trained with Q-backpropagation [101] and an SVM classifier [105], obtaining 90.91–92.19% and 98.8% accuracy, respectively. Consequently, the SVM classifier demonstrates similar performance with the two previous DL approaches.

Furthermore, GRF signals have fed ML models for differential diagnosis of several neurological disorders. Papavasileiou et al. [107] discriminate PD patients from post-stroke patients and from healthy controls with a multiplicative multi-task feature learning model, achieving 0.88–0.994 AUROC. Additionally, in [108], kNN, SVM and RF classifiers manage to discriminate PD patients from Huntington’s disease (HD) and amyotrophic lateral sclerosis (ALS) patients, as well as from healthy controls with 81.25–90.91% accuracy. The fact that the classification of PD patients and patients with other neurological disorders is a rather harder problem than the binary classification between PD patients and healthy controls is reflected in the obtained accuracies.

#### 3.2.3. Image and Depth Sensors

Useful information regarding the manifestations and the course of PD can also be acquired with the help of 2D or 3D video recordings or even images, in more rare occasions, after they are analyzed with computer vision techniques and ML methods. More specifically, this type of input data has been exploited for PD diagnosis, severity estimation, FoG and other symptom detection, medication state detection, segmentation of exercises and rehabilitation assessment. These insights have been extracted from 15 related studies presented hereafter, out of which 8 studies capture movements of the whole body while walking or exercising, 1 combines videos from several body parts, 4 focus on hand movements and 2 capture facial expressions. The respective findings are thoroughly presented in Table A3 in Appendix A of this paper.

Firstly, postural and kinematic features are extracted from videos that are recorded while PD patients and healthy controls are walking, to discriminate them. In [109,110], ANNs are trained with signals collected via the Microsoft Kinect (MS Kinect) sensors to diagnose PD. In [109], the combination of a CNN with an LSTM outperforms the stand-alone CNN and LSTM classifiers, reaching up to 83.1% accuracy, while in [110], the ANN classifier outperforms the SVM model, with 89.4% accuracy. In the last study, besides gait data, features extracted from finger- and foot-tapping activities have been evaluated, without however improving the classification performance. In the same vein, in [111], raw video-recorded gait sequences feed a CNN and discriminate PD patients from healthy controls with 88–90% accuracy. Finally, no studies that propose conventional ML algorithms as the best option to detect PD based on gait features extracted from videos were identified among the studies considered in the current systematic review.

Moreover, kinematic parameters from hand motor tasks can be extracted with the help of Leap Motion sensors and feed conventional ML algorithms to diagnose PD. For example, in [112], these signals train a bagged tree classifier, achieving 98.62% accuracy, while in [113], an SVM classifier demonstrates similar performance (98.4% accuracy) regarding PD diagnosis. Other tested algorithms that did not outperform the previous span across kNN, DT, LDA and LR models. Additionally, the authors in [114], train, again, an SVM model with automatically extracted features via CNNs and AEs from video recordings of hand movements to detect PD, obtaining 91.8% accuracy. They further classify PD patients with and without medication, obtaining a justified poorer performance (73.5% accuracy).

Furthermore, facial video recordings and images extracted from their frames discriminate PD patients from healthy controls as well. Firstly, in [115], an SVM classifier detects PD with f1-score = 99%, outperforming other conventional ML models and DL approaches, including recurrent neural networks (RNNs) and LSTMs. Additionally, in [116], only static information is provided to train DTs and RFs among other ML models, decreasing the obtained classification performance to 60.7–85.92% accuracy. It is obvious that data extracted from videos and not just static images can be leveraged to address PD-related problems more efficiently, as expected. Finally, in most cases hand features collected with the Leap Motion sensors diagnosed PD with higher accuracy than gait and facial features.

In addition, image and depth sensors can support the quantification of PD symptoms. In [110], PD severity is classified as mild or moderate with ANN or SVM models trained on gait, finger- or foot-tapping features extracted from MS Kinect. The highest classification accuracy is achieved with ANNs over gait data and reaches up to 95%. Similarly, a combination of two parallel LSTMs has classified UPDRS scores with 77.7% accuracy, based on gait-related MS Kinect signals [117]. On the other hand, Li et al. [118] estimate the exact values of UPDRS-III scores with an RF regressor based on 2D-videos, which recorded leg movements and toe tapping activities. The obtained RMSE is 7.765. In the same study, levodopa-induced dyskinesia severity in the Unified Dyskinesia Rating Scale (UDysRS-III) has been also estimated, this time based on video-recorded drinking and communication activities with RMSE = 2.906. Finally, Liu et al. [119] train SVMs with features extracted from video recordings of hand movements to classify bradykinesia-related MDS-UPDRS scores, achieving 89.7% accuracy. Regarding the task of severity estimation, in contrast to the diagnosis task, upper-limb analysis does not seem to be more insightful than lower-limb analysis.

In the end, some other PD-related problems that can be addressed with image and depth sensors are presented in this paragraph. Firstly, Timed Up and Go (TUG) tests have been video-recorded to shed light on several PD manifestations. In [120], the subtasks of a TUG test are automatically segmented with the help of an LSTM, obtaining 93.1% accuracy. Furthermore, in [121] an RNN with gated recurrent units (GRUs) is deployed to detect FoG episodes based on video-recorded TUG tests. The respective classification performance reaches up to 82.5% accuracy. Another task addressed based on signals acquired with MS Kinect sensors is the estimation of the adherence to medication [122]. DTs, RFs and SVMs achieve that with accuracy greater than 67.7%, when personalized models are built. Finally, in [123] a virtual physical therapist is developed. In this case, the subtasks of the performed exercises are automatically segmented with HMMs, the patients’ performance is estimated with an SVM classifier, which achieves 86.3–94.2% accuracy, and finally, the next steps regarding the next exercise to perform are recommended again automatically with RFs.

#### 3.2.4. Voice and Audio Sensors

Speech impairment is common among PD patients and could serve as an indicator of the presence and the degree of severity of the disease. Therefore, speech recordings, which can be usually collected with the help of smartphones or other portable devices, have been exploited for PD diagnosis, differential diagnosis and severity estimation, according to the respective eight studies considered in the current literature review. These studies and their main findings are presented hereafter and are also summarized in Table A4 in Appendix A of this paper.

In four of these studies, the detection of PD is discussed. Firstly, the authors in [124,125] leverage the vocal measurements gathered in the mPower study [126] to discriminate PD patients from healthy controls. In the first case, features are automatically extracted with the help of a CNN and the classification is performed with 90.45% accuracy, while in the second case features are manually extracted in time, frequency and cepstral domain and train a XGBoost classifier, after least absolute shrinkage and selection operator (LASSO) was applied, achieving 95.78% accuracy. Similarly, Zhang et al. [127] deploy stacked AEs to extract features from smartphone speech recordings. These features are forwarded to several ML algorithms, including SVM, kNN, NB, DT and LDA models. The kNN algorithm outperforms the others in the PD detection task with accuracy ranging between 94% and 98%. In the same vein, in [128] various types of features, such as phonation, unvoiced and speech are extracted from signals collected with smartphones and acoustic cardioid sensors. Again, the kNN model outperforms the others when it is trained with phonation features, achieving 92.94–94.55%. Consequently, one can observe that simple classifiers, such as the kNN and boosting algorithms, trained with vocal features usually outperform other more complex approaches in detecting PD. Finally, differential diagnosis between PD patients, rapid eye movement sleep behavior disorder (RBD) patients and healthy controls is performed with an RF classifier based on smartphone speech recordings [129]. Each health state is detected with a relatively low sensitivity ranging between 59.4% and 74.9% for each pairwise classification.

In the last study, the exact values of the scores in several PD-related scales are also estimated with an RF model trained over smartphone speech recordings. Low MAE is obtained for each one of the included clinical scales, which span across MDS-UPDRS, Montreal Cognitive Assessment (MoCA), Epworth Sleepiness Scale (ESS), Beck Depression Inventory (BDI) and Visual Analog Scale (VAS). Moreover, UPDRS scores have been estimated with approximately 2–3 MAE by a positive transfer learning model which makes use of speech recordings captured by a portable device [130]. The same data source is exploited in [131] for UPDRS score predictions in approximately 6 months. The XGBoost regressor achieves 5.09 MAE. Similarly, scores in the motor subscale of the UPDRS (mUPDRS) are estimated with ridge regression in [132] and are predicted in approximately 6 months with XGBoost regression in [131]. The obtained MAE are 5.5 and 6.45, respectively. As expected, the prediction of the course of the disease is harder than the current estimation of its severity. This is reflected in the slightly higher error metrics in the first case. However, in both cases, very low errors are obtained, in most cases with the help of ensemble ML techniques, such as bagging and boosting.

#### 3.2.5. Other Types of Sensors

Besides the aforementioned types of sensors, there are other smart devices that can collect signals to support PD patients’ diagnosis and monitoring. These signals may be related to muscles, brain or heart function, to body temperature or other motor-related measurements, such as displacement and keyboards dynamics. In this respect, eight related studies have been considered in this review, the details of which are presented in Table A5 in Appendix A of this paper. These studies are further presented below and address problems such as PD diagnosis, symptom detection and estimation of their severity levels, medication response estimation and emotion recognition.

Firstly, several devices that are widely used as conventional medical equipment can be adopted in free-living environments in portable versions as well. For example, EEG signals produced by portable headsets have trained feed-forward NNs to detect PD with 96.5% accuracy [133]. Surface EMG (sEMG) signals collected from wrist-bands have trained RF regression models that estimate MDS-UPDRS III scores with 0.739 correlation coefficient [134]. Additionally, Capecci et al. [135] have classified emotions as positive or negative with the help of SVMs trained over temperature, heart rate and galvanic response signals, which are acquired via a smartwatch. The respective classification accuracy ranges between 88.6% and 91.3%.

Furthermore, echo state networks (ESNs) have been trained over finger position sequences that were collected with finger-mounted electromagnetic sensors while performing finger-tapping tests to diagnose PD [136]. The respective AUROC reaches up to 0.802. Similarly, Picardi et al. [137] gather flexion measurements from smart gloves and orientation data from a wrist-worn tracking system and train SVM, ANN and genetic programming algorithms to classify PD patients and healthy controls with several levels of cognitive impairment. All the deployed algorithms demonstrate similar performance, with AUROC ranging between 0.72 and 0.99.

Useful features that shed light on several PD motor aspects can also be extracted from writing, drawing or typing activities on computer keyboards or tablet devices. In [138], spatiotemporal features are extracted while PD patients are drawing spirals on a touchscreen. These features train an MLP model that differentiates PD patients with bradykinesia from PD patients with dyskinesia, reaching up to 84% accuracy. Keystroke logs have also been exploited for PD diagnosis [139] and estimation of the medication response [140]. In both cases, DNNs have been deployed. In the first case, an LSTM with fuzzy recurrent plots outperforms CNNs, achieving 65.14% accuracy, while in the second case, an RNN achieves 76.5% classification accuracy and 0.75 AUROC. In the last study, the authors further predict the response to the prescribed medication in the future with 0.69–0.75 AUROC. As expected, the prediction task is harder than the estimation one, obtaining a lower AUROC.

#### 3.2.6. Combination of the Previous Sensors

There are also 22 studies that combine two or more types of sensors to address PD-related problems. Some commonly encountered combinations span across inertial and pressure sensors, inertial or pressure sensors with other biosensors, e.g., sEMG, heart rate and temperature portable devices, inertial or pressure sensors with speech or video recordings, among others. Some of the problems discussed in these studies are PD diagnosis, differential diagnosis, symptom detection and estimation of their severity, as well as FoG episodes and fall risk detection. More details on these approaches are presented in Table A6 in Appendix A of this paper.

More specifically, in some studies, position, inertial and pressure signals are fused to diagnose PD, detect its manifestations and quantify their severity. For example, Aharanson et al. [141] develop a smart support walker that contains two encoders on the wheels, two force sensors underneath the hand grips and a tri-axial accelerometer. They reduce the dimensionality of the derived signals with principal component analysis (PCA) and feed an LDA model to detect PD with 91–96% sensitivity. Inertial and in-sole pressure sensors have been also combined for FoG detection and prediction, by identifying the pre-FoG state [142]. Selected features in time and frequency domain train a RUSBoost classifier and detect or predict FoG events with 81.4–98.5% and 61.9–78% sensitivity, respectively. The results confirm that FoG prediction is a harder task than FoG detection. Moreover, Wu et al. [143] classify PD patients according to their UPDRS-III scores based on displacement and acceleration signals from hand movements. NNs outperform conventional ML models, such as SVMs and RFs, obtaining 91.18–95.30% accuracy.

Furthermore, there are three studies that combine acceleration and EMG signals to support PD patients at various aspects of the disease. Firstly, Cole et al. [144] leverage these signals to detect tremor and dyskinesia with the help of a HMM and a DNN, respectively, and estimate their severity levels with a Bayesian classifier. The severity of tremor is estimated with slightly higher sensitivity (95.2–97.2%) than the severity of dyskinesia (91.9–95%). Secondly, Hossen et al. [145] perform differential diagnosis between PD and ET patients with an MLP classifier, achieving 91.6% accuracy. Finally, Tahafchi et al. [146] detect FoG episodes with the help of a fully connected NN, achieving 0.906–0.963 AUROC. The results indicate that portable EMG devices can support FoG detection as effectively as pressure sensors when they are combined with IMUs.

Similarly, in [147], force, inertial and mechanomyography (MMG) signals train three distinct classifiers, kNN, MLP and AdaBoost, to diagnose PD and quantify its symptoms. More specifically, PD patients are discriminated from healthy controls with 96.6% average accuracy, PD patients with positive UPDRS scores are discriminated from those with zero due to DBS with 89% average accuracy and UPDRS scores levels are classified with 85.4% accuracy. Moreover, skin-mounted sensors with acceleration, gyroscope and thermometer measurements collect signals during TUG tests, which train CNNs for multi-source multitask learning to detect fall risk and estimate the severity of PD [148]. In the first case, a 94% f1-score is obtained, while in the second, 0.06 RMSE is achieved. In all these studies that make use of inertial or pressure sensors combined with other biosensors, PD severity is successfully estimated, with high accuracy in the classification case and low error in the regression.

Moreover, sometimes, signals from inertial sensors and speech recordings are combined to diagnose PD and quantify its severity. For example, an ensemble learner consisted of kNN, SVM and NB models detect PD with accuracy 99.8% when it is trained over phonation recordings and acceleration signals collected with a smartphone [149]. In the same study, MDS-UPDRS levels are classified with a kNN model, achieving 90.5–98.5% accuracy. Similarly, in [150], accelerometer, gyroscope and magnetometer data are combined with speech recordings acquired from a headset and train an extreme learning machine (ELM) that classifies PD patients at several severity levels and healthy controls with accuracy ranging between 92.45% and 95.93%. There is no significant difference between the obtained accuracies, regarding the severity classification of these two approaches. On the other hand, Papadopoulos et al. [151] combine accelerometer with typing dynamics data collected from a smartphone to detect tremor or fine-motor impairment and finally diagnose PD by training NNs, achieving 0.834–0.868 AUROC.

Moreover, inertial or pressure measurements are usually used together with video recordings or images to feed ML algorithms and diagnose PD. For example, in [152], acceleration signals and silhouette images are collected during cooking. Convolutional variational autoencoders (VAEs) are deployed to extract features that will feed NNs to finally differentiate PD patients from healthy controls with 66% f1-score. Similarly, Wahid et al. [153] diagnose PD with the help of an RF classifier trained over spatio-temporal gait features extracted from videos and GRF signals. The respective AUROC reaches up to 0.96. Albani et al. [154] exploit videos that record upper-limb movements for pretraining and then lower-limb motor signals from IMUs to train a kNN that detects PD with 91.5–98.6% accuracy and estimates its severity according to the UPDRS with 60.7–79.1% accuracy. It is observed that RF and kNN models detect PD more accurately than NNs, which are deployed in [152]. Visual and audio features can also be extracted from short interview audio-video clips to train an NN that will estimate facial expressivity of PD patients [155]. A low f1-score (55%) is obtained in the classification approach, while MAE = 0.48 is obtained in the regression approach.

Additionally, there are a few studies that take advantage of smart pens equipped with various types of sensors. All of them differentiate PD patients from healthy controls by training either conventional ML algorithms or DNNs. In [156], an RF classifier is proposed which is trained with the most important principal components of pressure, tilt and acceleration signals. In this approach, PD is detected with 88.8–89.4% accuracy. Similarly, Gallicchio et al. [157] diagnose PD with an ensemble model of deep ESNs trained with pen position, pressure and grip angle signals, achieving 89.33% accuracy. The aforementioned signals along with recordings from a microphone embedded in the smart pen have also been fed to either a pre-trained CNN or an optimum path forest (OPF) classifier and PD is successfully detected with 77.92–87.14% accuracy [158]. To conclude, all these approaches demonstrate similar performance in diagnosing PD. Only the OPF model seems to perform slightly poorer than the rest methods. Finally, data acquired from a smart pen can be combined with shoe-mounted IMU signals and train an AdaBoost classifier that manages to detect PD with sensitivity up to 100% [159].

There are also two studies [160,161] that use multiple types of data acquired with the help of a mobile application in the mPower study [126]. These data include touchscreen logs from a tapping activity, accelerometer signals from a walking activity, performance in a memory game and vocal measurements. In both studies, ensemble methods that combine DL methods, such as CNNs and RNNs, and conventional ML approaches, such as LR and RF, have been deployed to detect PD with f1-score 82% and 87.1%, respectively. Finally, in [162], several data have been collected from ambient sensors in a smart house environment, including infrared motion sensors on the ceiling, light sensors, magnetic door sensors, temperature sensors and vibration sensors on selected items, along with signals from two wearable inertial sensors. All these data after dimensionality reduction with either k-means or random resampling were used to train an AdaBoost classifier that diagnoses PD with 80–86% accuracy.

## 4. Collective Comparison and Overall Insights across the Studied Approaches

The literature review presented herein considers 112 research studies that leverage IoT technologies and ML methods to support neurologists at various decision-making processes involved in every stage of PD and thus potentially advancing PD patients’ care and quality of life. The considered approaches are grouped per type of sensor used for data collection. For each individual study, the problem addressed, the dataset used for training and evaluation, the input data, the processing techniques and the AI algorithms deployed, as well as the evaluation metrics and the final performance of the proposed models are briefly presented. Different approaches within each category of type of sensors are compared with each other, in terms of performance, to finally infer which ML algorithms are most suitable to address the discussed problems with respect to the specific type of input data, each time. After presenting the results, additional pieces of evidence should be provided related to the obtained average performances in the scope of both intra- and inter-categories of deployed sensors, to identify ill-addressed problems and further infer which type of input data may be more insightful to address each of the discussed PD-related problems.

### 4.1. Sensors and Devices Deployed

**Figure 3:**
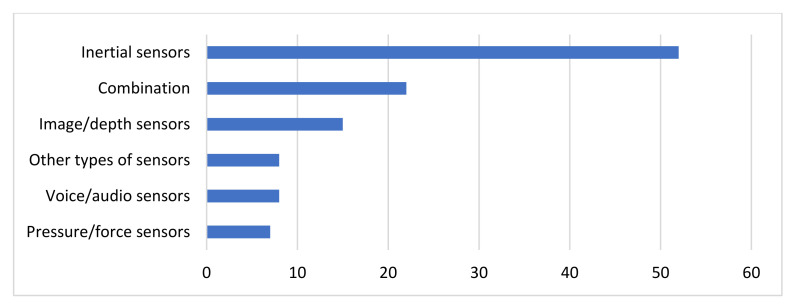
Distribution of the deployed types of sensors over the considered studies.

The types of sensors that are deployed for data collection and their popularity among the studies considered are depicted in Figure 3. As Figure 3 indicates, inertial sensors or IMUs, including accelerometers, gyroscopes and magnetometers are by far the most popular data source among the considered studies. Combination of different types of sensors follow in popularity, where again most of the times one from the multiple data sources used corresponds to inertial sensors. Moreover, data collected with image or depth sensors are another popular type of input data. On the other hand, speech recordings collected with audio or voice sensors and force signals collected with pressure sensors are more rarely encountered in the literature. Finally, it is worth mentioning that despite EEG, EMG and other medical signals are commonly used in the clinical practice for PD diagnosis, severity estimation and course prediction, there are very few studies considered that make use of portable biosensors, which monitor brain, heart or muscles functionality. These types of sensors fall in the less popular category, named as other, which includes besides portable EEG, sEMG and ECG devices, thermometers, galvanic response sensors, as well as flexion sensors, position sensors, encoders, tracking devices, time loggers, ambient sensors, including light, temperature, vibration sensors, etc.

Additionally, the systematic review presented in this paper is focused on IoT approaches, which means that all the aforementioned sensors must be embedded in devices that can transfer the collected patients’ data to other computational machines or remote databases via Wi-Fi, Bluetooth or any other communication protocol. To this end, the discussed types of sensors may be embedded in smartwatches, smart bracelets, shoes’ insoles, other wearable devices, smartphones, video cameras, other portable devices and other everyday objects in general.

**Figure 4:**
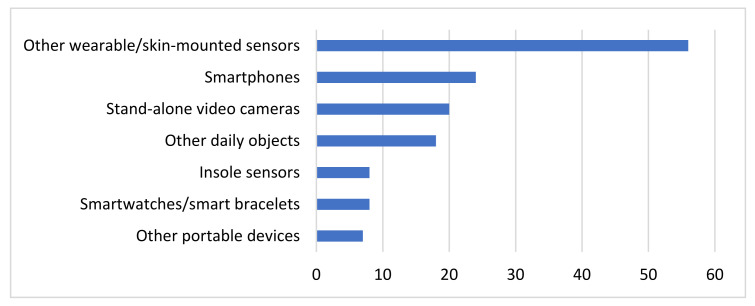
Distribution of different devices deployed over the considered studies.

As Figure 4 indicates, the most widely encountered type of device in the literature corresponds to other wearable sensors or sensors that are mounted somehow to the patients’ corpus, e.g., via flexible bands, such as Shimmer3 or Physilog sensors. Mostly IMUs are embedded to this type of devices, but there are some other types of sensors and one reference to a pressure sensor that were embedded to these devices among the studies considered. The second most popular type of devices deployed is the smartphones. The smartphones are leveraged for their in-built sensors, which include but are not limited to IMUs, microphones, cameras and others. Stand-alone video cameras follow in popularity, including the Microsoft Kinect tool and the Leap Motion sensor, which are mainly used for video recordings but were also used once for speech recordings as well. Moreover, in 18 studies everyday objects that do not fall into other categories were deployed. For the specific studies considered in this review, these objects span across smart pens, keyboards, Wii controllers, support walkers and spoon handles. Various types of sensors, including inertial, pressure, audio and other are embedded to these objects. On the other hand, insole sensors are deployed solely for collecting gait-related pressure signals from the respective eight studies presented in Section 3.2.2. Similarly, smartwatches and smart bracelets are exploited almost exclusively for collecting inertial signals. Finally, in a few studies some other portable devices are used for speech recordings and other measurements.

### 4.2. Meta-Analysis over Sensor-Specific Results Obtained

For each category of sensors, the problems addressed and the respective average obtained accuracies are presented. Each subsection dedicated to one type of sensor is accompanied by one column chart which summarizes which problems are addressed based on the respective type of sensors and one boxplot that illustrates the performance range achieved for each problem addressed. Boxplots are selected for the latter cause as they offer an excellent illustration of the dispersion of the data. More specifically, the mean of the values is depicted by an ‘x’ and the median by a horizontal line which divides the data points in a top and a bottom half. The medians of these two halves form a box which corresponds to the interquartile range. The values outside this range are depicted by two whiskers (vertical lines), and there may also be some outliers, as distinct data points that exceed 1.5 times or more the interquartile range.

#### 4.2.1. Inertial Sensors

Based on the results presented in Section 3.2.1, it is evident that inertial sensors have already been widely used in the literature to address various PD-related problems with the help of ML and DL algorithms. Diagnosis and differential diagnosis are two commonly addressed problems of this category. Diagnostic models demonstrate very high performance, with approximately 88.5% average accuracy and 89.5% median accuracy, while the performance of differential diagnosis models is a bit poorer, with accuracy ranging between 72% and 89%. However, if one takes into account that it is much more difficult to differentiate patients with similar manifestations, the obtained performance can be considered very satisfactory.

Other popular problems addressed based on the usage of inertial sensors are the detection and the quantification of PD symptoms. Tremor, bradykinesia and levodopa-induced dyskinesia detection corresponds to a classification problem, for which approximately 84.5% average accuracy is obtained from the considered studies. FoG episodes seem a little easier to be detected, as the average accuracy for this classification task is approximately 90%. Regarding the severity estimation of PD symptoms, the classification approach is presented in more studies than the regression and the respective average accuracy (approximately 83.5%) is a bit lower than the accuracies obtained in the two previous problems, which can be attributed to the increase in targets-classes. Finally, the detection of medication-related fluctuations and the estimation of the patients’ response to medication are some problems that are addressed more rarely based on data collected with inertial sensors. The respective average accuracy is again very satisfactory but remains the lowest achieved (77%) regarding the detection of fluctuations among all the other problems. Finally, identification of PD subtypes, gait segmentation and rehabilitation assessment are some problems that are addressed only once among the studies considered based on inertial signals.

**Figure 5:**
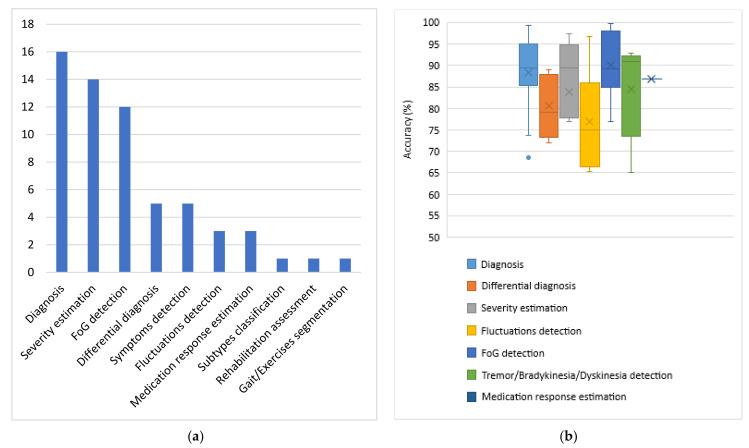
PD problems addressed based on inertial sensors and the respective obtained accuracies. (**a**) Number of studies per task; (**b**) accuracies obtained from the proposed models for each category of addressed tasks. Number of studies considered in this boxplot per task: *n* = 12 for diagnosis, *n* = 6 for differential diagnosis, *n* = 8 for severity estimation, *n* = 3 for fluctuations detection, *n* = 11 for FoG detection, *n* = 4 for tremor/bradykinesia/dyskinesia detection and *n* = 1 for medication response estimation.

The occurrences of all the problems tackled with inertial data are summarized in the column chart of Figure 5, which confirms that PD diagnosis, symptom severity estimation and FoG detection are the most widely addressed problems based on inertial data. A wide variety of ML and DL algorithms have been proposed for all these cases. Moreover, the obtained range of performances for each category of tasks is illustrated in the boxplot of Figure 5, based on the accuracy metric, which is the simplest and most widely used, among the considered papers, evaluation metric. Studies that do not apply this metric in their evaluation stage are not considered in the boxplot of Figure 5. Consequently, the construction of this boxplot is based on 45 discrete models out of the 52 inertial sensors-based studies, which are considered in this review and for each one of them only the best performing proposed method is taken into consideration.

#### 4.2.2. Pressure and Force Sensors

**Figure 6:**
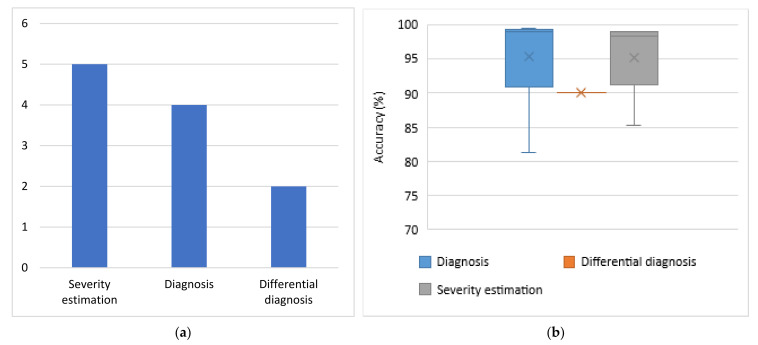
PD problems addressed based on pressure sensors and the respective obtained accuracies. (**a**) Number of studies per task; (**b**) accuracies obtained from the proposed models for each category of addressed tasks. Number of studies considered in this boxplot per task: *n* = 4 for diagnosis, *n* = 1 for differential diagnosis and *n* = 5 for severity estimation.

As the column chart of Figure 6 indicates, very few studies that address solely PD diagnosis, estimation of the respective severity and differential diagnosis based on pressure sensors have been identified across the considered studies. No force sensors-based studies that address the problems of FoG detection, PD symptoms and their fluctuations detection, as well as medication response estimation were identified. Moreover, as the boxplot of Figure 6 indicates, when pressure or force sensors are deployed, PD diagnosis is performed with approximately 95% average accuracy and 99% median accuracy, severity estimation is performed again with approximately 95% average accuracy and 98% median accuracy, and differential diagnosis is performed with slightly lower accuracy (90%). Again, only the studies that make use of the accuracy metric for the evaluation of the proposed models are taken into consideration for the construction of this boxplot. This time, only one out of the seven studies discussed in this category is excluded.

#### 4.2.3. Image and Depth Sensors

**Figure 7:**
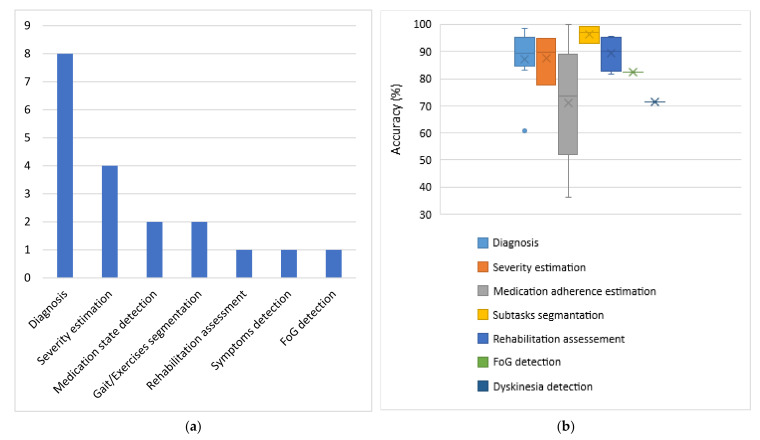
PD problems addressed based on image and depth sensors and the respective obtained accuracies. (**a**) Number of studies per task; (**b**) accuracies obtained from the proposed models for each category of addressed tasks. Number of studies considered in this boxplot per task: *n* = 7 for diagnosis, *n* = 3 for severity estimation, *n* = 2 for medication adherence estimation, *n* = 2 for subtasks segmentation, *n* = 1 for rehabilitation assessment, *n* = 1 for FoG detection and *n* = 1 for dyskinesia detection.

As the column chart in Figure 7 indicates, PD diagnosis is the most commonly addressed problem among the considered studies, based on input data collected from image and depth sensors, while estimation of PD symptom severity follows. In more rare cases, this data source has been used to detect medication state, segment exercises and motor tests and assess rehabilitation, as well as to detect FoG episodes and other PD-related symptoms. It is also worth mentioning that no studies addressing the problem of differential diagnosis based on this type of sensors were identified. Consequently, a reasonable question raised is whether video recordings can differentiate patients with similar manifestations. Moreover, although computer vision has been widely used for mood estimation of healthy people, no studies were identified to address this problem based on PD patients’ data.

Finally, the obtained accuracies for each category of addressed PD-related problems based on video recordings or imagery input data are depicted in the boxplot of Figure 7. As 2 out of the 15 studies discussed do not measure the performance of the proposed methods with the accuracy metric, they are excluded from this boxplot. By taking into consideration the remaining studies, the following observations can be made. The best performance (96.5% average accuracy) is achieved for the segmentation of exercises and motor clinical tests in subtasks. Rehabilitation assessment tasks are also effectively performed based on video recordings, with 89.5% average accuracy. PD diagnosis and severity estimation follow with average accuracy slightly greater than 87%. Finally, ML algorithms perform a bit poorer in detecting FoG episodes (82.5% accuracy), dyskinesia (71.4% accuracy), as well as in estimating medication adherence (71% average accuracy), when images and videos are used as inputs.

#### 4.2.4. Voice and Audio Sensors

Based on the results presented in Section 3.2.4, speech signals have been exploited for PD diagnosis in four studies, for quantification of symptom severity in four studies and for differential diagnosis in only one study. No studies that address the problems of fluctuations detection and medication response estimation were identified. Every study in this category that addresses the symptoms quantification problem proposes a regression and not a classification model. Consequently, their performance cannot be compared with the performance obtained in the diagnosis task, in terms of the obtained accuracy. Moreover, the accuracy metric is not used in the evaluation of the differential diagnosis which is performed in [129]. Therefore, only the accuracies of ML algorithms regarding PD diagnosis from four studies could be compared with each other. In all these cases, the obtained performance is very high, with accuracy greater than 90% (approximately 94% average accuracy). Due to the lack of more accuracy data points, the presentation of the respective boxplot diagram, in alignment with the previous subsections is considered to be unnecessary.

#### 4.2.5. Other Types of Sensors

Moreover, various PD-related problems, such as diagnosis, detection of symptoms and estimation of their severity, estimation of patients’ response to the prescribed medication and emotion recognition, are addressed based on input data provided by a wide variety of other sensors. Due to this variability, it is considered that there is no reason to proceed to a grouping of approaches or to a thorough comparison between the different considered studies. The only common addressed problem is the diagnosis of PD and the highest accuracy for this task is obtained when EEG signals are used. Approaches based on finger-mounted position sensors and hand-mounted flexion sensors follow. Finally, the lowest accuracy is obtained when keystroke log data are deployed.

#### 4.2.6. Combination of the Previous Sensors

**Figure 8:**
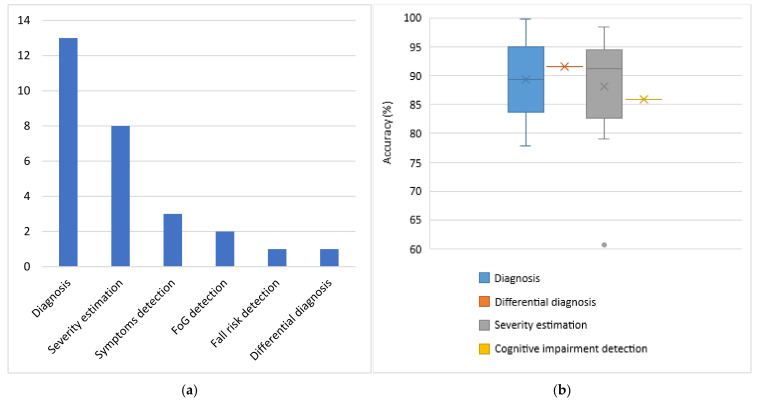
PD problems addressed based on data collected via a combination of various types of sensors and the respective obtained accuracies. (**a**) Number of studies per task; (**b**) accuracies obtained from the proposed models for each category of addressed tasks. Number of studies considered in this boxplot per task: *n* = 9 for diagnosis, *n* = 1 for differential diagnosis, *n* = 5 for severity estimation and *n* = 1 for cognitive impairment detection.

As the column chart of Figure 8 indicates, the combination of more than one different type of sensor has been exploited mainly for PD diagnosis and symptom severity estimation, and more rarely for FoG and other PD symptom detection, fall risk identification and differential diagnosis. Moreover, no studies that address the problems of fluctuations detection and medication response estimation were identified. Additionally, the obtained accuracies for each category of addressed PD-related problems based on fusion of data obtained by combinations of various types of sensors are depicted in the boxplot of Figure 8. The main classification tasks, which are evaluated based on the accuracy metric, are PD diagnosis, differential diagnosis, severity estimation and cognitive impairment detection. Therefore, 13 out of the 22 total studies, which address these problems, are considered in this boxplot. In all these cases, features extracted from multimodal sensory data lead to very satisfactory results. PD is detected by an average accuracy of approximately 89%. On the other hand, PD symptoms are quantified with approximately 88% average accuracy and 91% median accuracy. Consequently, despite severity estimation is a hard problem, it can be addressed with the help of various sensory data to a sufficient extent, with an approximately equal performance to the one achieved in the diagnosis case. Finally, differential diagnosis demonstrates 91.6% accuracy, cognitive impairment detection demonstrates 86% accuracy, and the remaining tasks are evaluated based on other metrics and therefore are not considered in the boxplot of Figure 8.

### 4.3. Overall Addressed Problems Related to Parkinon’s Disease and Obtained Accuracies

#### 4.3.1. Frequency of Problems Addressed

**Figure 9:**
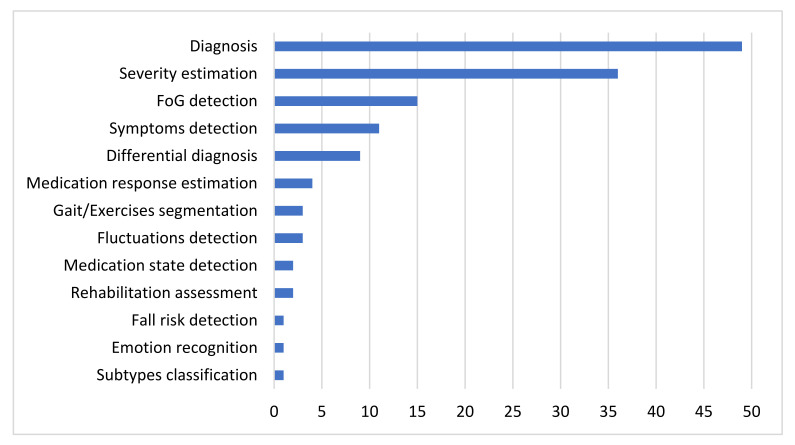
Frequency of PD-related problems studied independently of type of input data used.

As Figure 9 indicates, diagnosis is the most widely addressed problem among the considered studies and corresponds to a binary classification problem between PD patients and healthy controls. The quantification of PD symptom severity follows, which can be both a classification and a regression task, most of the time with respect to specific scales, such as the UPDRS and the H&Y scale. However, most of these approaches estimate the current patients’ situation and do not make any predictions in the future, which would be more fruitful for personalized treatment suggestions. The detection of FoG episodes and other PD symptoms, including tremor, bradykinesia, dyskinesia, cognitive impairment and deficient facial expressivity, are also quite popular topics in the current literature search. These are binary classification problems between the presence and the absence of certain manifestations. Differential diagnosis follows, which corresponds to either a binary or a multi-class classification problem between PD patients and patients with other neurological disorders.

Despite fluctuations detection, medication state detection, estimation of patients’ response to medication and rehabilitation assessment are problems of utmost importance to achieve precise medicine, they are encountered more rarely in the literature. Most of the times, these approaches correspond to classification problems, but they can also be seen as regression problems, in the terms of, e.g., specific treatment indices estimation. Additionally, gait and exercises segmentation, which can be addressed by both supervised and unsupervised learning techniques, is presented in few studies. Finally, some other classification problems, which are discussed only once across the studies considered in this paper, include fall risk detection, emotion recognition and PD subtypes identification. All these findings are summarized in the bar chart of Figure 9.

#### 4.3.2. Overall Performance Comparisons per Task

Firstly, diagnosis, which is the most common addressed problem, is performed based on input data produced from all the types of sensors discussed. In every case, classification results are very satisfactory and average accuracy is greater than 87%. The highest average accuracy is obtained for studies based on pressure signals (approximately 97% accuracy) and then studies based on voice recordings. Other types of sensors and combinational analyses follow. Finally, approaches based on inertial, image and depth sensors perform slightly worse, with approximately 87% average accuracy.

Additionally, severity levels are again more accurately estimated by pressure signals (95% average accuracy). Video recordings and combinations of different types of sensors follow, while approaches based on inertial signals achieve 85% average accuracy. Moreover, despite severity estimation is performed based on data produced from all the types of sensors discussed, speech recordings and other types of sensors are solely deployed for PD symptom severity regression and not classification. Therefore, the performance of the respective models cannot be compared with the others, in terms of accuracy. Generally, the classification of PD patients to severity levels-categories, with respect to various motor and non-motor symptoms, is a more difficult problem than PD detection. This is reflected in the slightly lower accuracies obtained in the first case, in almost every category of sensory data and it can be attributed to both the increase in similarity among the classes-targets and the increase in the number of the classes.

**Figure 10:**
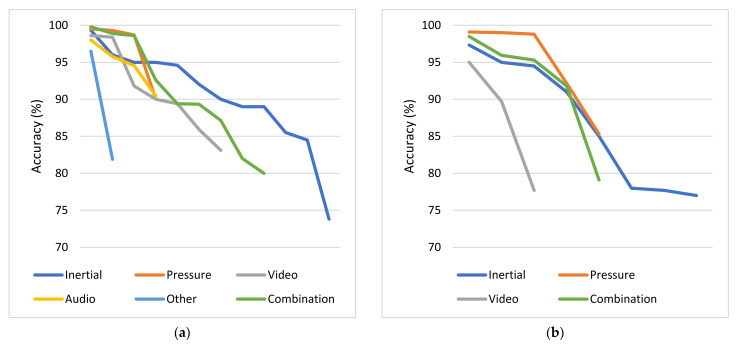
Comparison between the best performing proposed models among all the different types of sensors (**a**) regarding diagnosis; (**b**) regarding severity estimation. In both cases, the horizontal axis refers to the indices of the studies.

The highest obtained accuracies from all the proposed methods per category of sensors for these two popular tasks (PD diagnosis and severity estimation) are depicted in Figure 10. These diagrams confirm that inertial sensors are more widely exploited in the literature compared to other types of sensors for both diagnosis and severity estimation. Moreover, one can easily observe that independently of the type of sensors leveraged to collect input data, there are some ML or DL models that reach almost excellent classification accuracy (greater than or equal to 95%) with respect to both problems. The respective average accuracies range between 87% and 97%, demonstrating the predictive power of the hidden information of all the types of sensory data considered in the current literature review.

Another interesting question is whether the fusion of different types of data improved the respective performance of models trained on single data sources. Combinational analyses are mainly proposed for PD diagnosis and severity estimation. In both cases, it is easily observed that the average accuracy increased slightly when different data sources were combined to address these problems. However, this is not a statistically significant increase. Finally, as already mentioned, pressure sensors have led to higher average accuracies in diagnosis and severity estimation tasks than inertial sensors. However, this may be misleading, as there are many more inertial sensors-based studies which make use of many different datasets, while most pressure sensors-based studies leverage the same dataset. Therefore, the results obtained by the inertial sensors-based studies may be more stable and trustworthy.

Continuing with the remaining less frequently addressed problems, FoG episodes and other PD symptoms are detected almost exclusively by leveraging inertial sensors. In the first case, 90% average accuracy is obtained with inertial sensors, while there is also one video-based approach which detects FoG with slightly lower accuracy. In the second case, PD symptoms are detected with 85% average accuracy with inertial signals, while there is also a video-based approach which achieves slightly lower accuracy and an approach based on other types of sensors which demonstrates similar average accuracy. Finally, the exploitation of more than one types of sensors has been explored in both cases. However, the respective results are not evaluated with the accuracy metric, thus their performance is not compared with the inertial-based and video-based approaches.

Moreover, differential diagnosis is performed with every category of sensors presented, except for video recordings and other types of sensors. Most studies addressing this problem are based on inertial or pressure sensors. In concordance with the binary classification problem of diagnosis, the highest average accuracy (approximately 88%) is obtained when force sensors are deployed. Generally, classification performance for differential diagnosis is significantly lower than what is obtained for discrimination of PD patients and healthy controls. This can be attributed to the similarity among manifestations of different neurological disorders, as well as to the increase in the number of targets-classes, in some cases.

Finally, some other medication-related problems, such as the estimation of patients’ response to medication, the detection of symptoms fluctuations and whether a patient is on or off medication, are addressed with the help of inertial, image, depth and other sensors. No studies based on pressure or voice sensors were identified for this cause. The performance is slightly higher (88% average accuracy) for medication response estimation, when inertial sensors are used. Regarding fluctuations and medication detection, all the types of the deployed sensors demonstrate similar performance, with average accuracy ranging between 77% and 81%. Consequently, it can be concluded that this is a slightly harder problem than the ones discussed so far. In contrast, the segmentation of walking tests and other physical exercises to subtasks and phases, as well as the assessment of patients’ rehabilitation have been performed with exceptionally high average accuracy (96% and 90%, respectively), by video recordings. There is also one inertial sensors-based study which address the problem of gait segmentation, but the accuracy metric is not applied in the evaluation process.

### 4.4. Ranking of Machine Learning Algorithms

**Figure 11:**
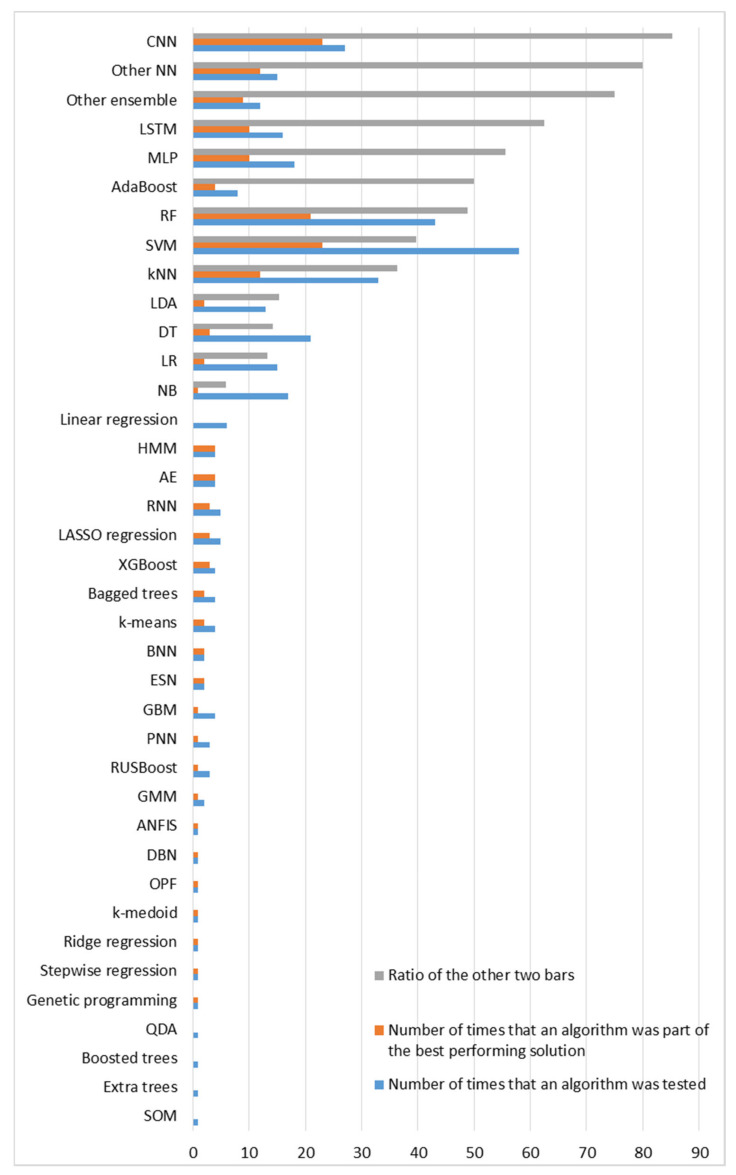
Distribution of the deployed ML algorithms over the considered studies. The blue bar depicts how many times each algorithm was tested, while the orange bar depicts how many times the same algorithm was selected as the best performing model or as a part of the final proposed solution. For the most widely tested algorithms, the ratio of the two previous values (grey bar) is also depicted (%).

All the considered approaches take advantage of AI, ML and DL algorithms, to predict, estimate or infer the desired results in each respective task. In Figure 11, all algorithms encountered in this systematic literature review are gathered. These algorithms correspond to classification, regression, clustering or simply automatic feature extraction and modelling techniques. The considered approaches span across probabilistic methods, such as NB, probabilistic neural networks (PNNs), ANFIS, HMMs, Bayesian neural networks (BNNs) and DBNs; simple conventional ML algorithms, such as LR, LDA, quadratic discriminant analysis (QDA), kNN, OPF, DTs and SVMs; ensemble learning methods, such as RFs, AdaBoost, RUSBoost, XGBoost, GBMs, bagged trees, boosted trees and extra trees, among others; and neural networks, such as MLPs, CNNs, LSTMs, RNNs, ESNs, self-organizing maps (SOMs) and AEs, among others. Many of the previous algorithms are used for both classification and regression tasks. Some other proposed models are suitable only for regression, including linear, ridge, LASSO and stepwise regression. Additionally, some clustering techniques used include k-means, k-medoid algorithms and GMMs. Finally, a genetic programming approach has also been discussed in one study.

As the blue bar indicates in Figure 11, the three most commonly evaluated models are SVMs, RFs and the kNN algorithm. CNNs follow, along with DTs, NB, LR, LDA models, MLPs, LSTMs, other NNs and other ensemble methods. The rest algorithms are encountered more rarely in the literature to address PD-related problems. The fact that these algorithms were discussed in many of the considered approaches does not necessarily mean that they were considered in the final proposed solutions, as the best performing models. The most commonly proposed algorithms (orange bar in Figure 11) are CNNs, SVMs and RFs, while the kNN algorithm, other NNs, MLPs, LSTMs and other ensemble methods follow. Indeed, although some simple models, such as DTs, NB, LR and LDA, are widely tested, they are rarely selected as the best performing solution when other, possibly more complex, algorithms are also tested in the same study. Regarding the respective ratio, CNNs, other NNs and ensemble methods demonstrate far better efficiency, as they are selected as the best performing solution in 85%, 80% and 75%, respectively, of the occasions that they are evaluated in. LSTMs, MLPs, AdaBooost and RFs follow, while SVMs and kNN demonstrate lower efficiency ratio. The rest methods either demonstrate even lower efficiency or are not taken into consideration in the ratio-based sorting, as they have been tested very few (equal or less than 5) times in the literature and the obtained results would be misleading and would lack credibility. Finally, it is worth mentioning that more and more complex DL and ensemble learning approaches are mainly proposed in the last few years.

### 4.5. Subjects Enrolled

**Figure 12:**
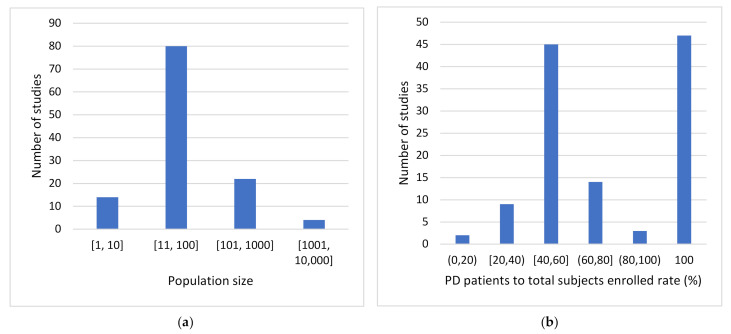
Distribution of the number of the enrolled subjects across the studies considered. (**a**) Total population size across the studies considered in logarithmic scale-based bins. (**b**) The rate of PD patients to total subjects enrolled across the studies considered in 20-width bins.

Another aspect which affects largely the obtained performance of the proposed ML models is the number and the variability of the subjects enrolled in each study. In the current review, most of the considered studies enroll a relatively small group of PD patients, healthy controls and patients with other neurological disorders. As the first column chart of Figure 12 indicates, the vast majority of the considered approaches enroll 10–100 subjects. Few studies enroll 100–1000 subjects and less than or equal to 10 subjects and only four studies enroll more than 1000 subjects. Moreover, in 47 approaches solely PD patients are enrolled, while in the rest of the approaches both PD patients and healthy controls or patients with other neurological disorders are enrolled to enable a differentiation between these groups. As the second column chart of Figure 12 indicates, in most cases, the classes of PD patients and other subjects are balanced, with PD patients rate ranging between 40% and 60%. When datasets are unbalanced, PD patients outnumber other groups in slightly more cases. The opposite is usually observed when large cohorts are exploited with more than 100 subjects enrolled. It is also worth mentioning that in six approaches, the problem addressed refers to a multiclass classification, where PD patients’ rate is usually much lower than 50% of the enrolled subjects, as expected. To conclude, despite the relatively small population size in most cases and the possible imbalanced classes in some cases, ML and DL models generalize reasonably well.

## 5. Discussion and Conclusions

The systematic review presented in this paper considers 112 studies, published in the last decade, which propose ML models trained with data collected via sensors and IoT technologies to address various PD-related problems. PD is a neurodegenerative disease that affects many, mainly elderly, people worldwide. The studies considered in this review provide evidence that useful knowledge can be extracted from sensory data with the help of AI methods, regarding motor and non-motor manifestations of PD, enabling doctors to make evidence-based decisions. This could potentially support personalized treatment suggestions and ensure high quality PD patients’ care even remotely. Thus, easing the burden of the healthcare system and moving from a clinic-centric to a patient-centric approach could be achieved. Therefore, it is crucial to summarize some early achievements obtained in this domain and identify remaining challenges and steps to be made in this direction.

Based on the results obtained by the presented review, it is concluded that inertial sensors have already been studied in depth for PD diagnosis and monitoring, while other less frequently used include image/depth, voice and pressure sensors, among others, as well as their combination. Moreover, there are indications that portable medical devices monitoring brain, heart and muscle functionality or other bio-signals, such as body temperature, have not yet been studied to a sufficient extent. Similarly, ambient sensors were encountered extremely rarely in the literature. It is very important that more experiments, exploiting other types of sensors rather than IMUs should be performed to address various PD-related problems.

Furthermore, the deployed sensors in the majority of the considered studies were not embedded in everyday objects and portable devices that patients are familiar with. Consequently, the need to explore the deployment of more everyday devices and wearables, such as smartphones and smartwatches among others is highlighted, to ensure unobtrusiveness and minimize the risk of PD patients’ stigmatization during the experiments. The wider deployment of these sensors is also expected to facilitate the execution of experiments in free-living environments with the help of IoT infrastructures. The vast majority of the considered studies were performed in laboratories or clinical environments and PD patients performed instructed movements. Only a handful of approaches examined the predictive power of sensorial data under free-living conditions. However, moving in this direction is of utmost importance, as sensors and IoT technologies need to be evaluated under real-world conditions, to finally achieve PD diagnosis and care revolution.

Additionally, to get more accurate measurements, the optimized position of each sensor needs to be identified. It is inferred from the considered studies that more accurate gait features were extracted when inertial sensors were mounted to the feet than when mounted to the waist. Similarly, the optimized arrangement of multiple sensors needs to be determined to maximize knowledge extraction and deploy methods for efficient multimodal data combination. Furthermore, sampling frequency should be fine-tuned in order to avoid scaling issues and minimize power consumption [163], while at the same time ensuring there is no significant information loss. Another crucial issue is the fast data transmission to remote databases and cloud servers, which may need to be performed even in real time, in some applications. The commercialization of 5G networks and the development of 6G networks prototypes is expected to solve this problem [164].

Moreover, PD-related problems addressed by the studies considered in this review include diagnosis, differential diagnosis, severity estimation, detection of FoG and other PD manifestations, fluctuations detection and medication response estimation, among others. PD diagnosis and estimation of symptom severity levels are the most in-depth studied problems, addressed with every type of sensor discussed. However, in the vast majority of the considered studies the current PD patients’ state was estimated and only in a handful of them, the course of the disease was predicted in the future. These predictions could actually revolutionize PD care by offering neurologists useful insights in advance and enabling them to act proactively, by, e.g., increasing the proposed levodopa daily dosage. Therefore, researchers should focus more on conducting such experiments based on various types of sensors. Additionally, other tasks such as fluctuations detection, medication response estimation and PD subtypes identification, which are of utmost importance to shed light on inter- and intra-patient variability and hence proposing personalized treatments, are addressed extremely rarely based almost exclusively on inertial signals. Consequently, it is highlighted that more experiments should be conducted in this direction. Finally, across the studies considered, specific popular tasks were not addressed at all by specific types of sensors, such as differential diagnosis based on video recordings. In this respect, it would be interesting to explore various types of sensors in new PD-related problems.

In principle, it can be concluded that very good results have already been achieved for all the discussed PD-related problems with the proposed AI methods based on each type of the deployed sensors. Some of these tasks can be addressed more easily and satisfactorily than others. For example, higher accuracy is obtained for diagnosis rather than differential diagnosis, prediction of symptom severity and fluctuation detection. Similarly, some types of sensors tend to lead to more accurate estimations, with respect to specific tasks, but that is also dependent on the specific datasets used, especially when few studies are considered in some categories of sensors. It is worth mentioning that in most cases, the combination of multiple types of sensors did not lead to a statistically significant increase in the obtained performance.

In any case, the most common best performing ML models correspond to CNNs, LSTMs, MLPs, other NNs, as well as ensemble learning techniques, such as boosting and RFs. It is clear that more complex approaches based on ANNs and metaclassifiers usually outperformed the simpler ones. The simpler approaches that follow are SVMs and the kNN algorithm. Moreover, the novel technique of transfer learning, which was discussed in a handful of the studies considered could be further tested to investigate whether it could support DL approaches and ensemble methods to obtain higher accuracies in PD-related problems, while at the same time it could potentially decrease training time. Additionally, in the vast majority of the studies considered, supervised approaches were discussed and only extremely few of them corresponded to semi-supervised approaches. Since a shift to collect patients’ data in free-living environments is highly suggested and that would indispensably lead to a large volume of unlabeled data, it is of utmost importance to follow more semi-supervised and self-supervised approaches that enable the exploitation of unlabeled data as well.

Moreover, to enable the exploitation of DNNs and recent advances in the big data era to address PD-related problems, larger cohorts of PD patients should be enrolled. In this respect, researchers should be encouraged to share the acquired data with the research community, to potentially enable the creation of data lakes with respect to PD. Furthermore, it would be useful to build both generalized and personalized models to support optimized predictions and estimations for new users-patients and patients who have already provided abundant data as well. In this direction, special care should be taken to maintain long-term patients’ databases and to leverage the digitalization of medical records. Finally, during every stage of the respective pipeline, very strict security guidelines should be followed to ensure patients’ data privacy and security. The advances in distributed systems and blockchains could support this cause [165].

To conclude, there is evidence that ML models and IoT technologies can revolutionize the way that PD and other chronic diseases are diagnosed and treated. As the promising results of the last decade indicate, the adoption of smart technologies in clinical practice can support clinicians in several decision-making processes, potentially reducing the current extremely high healthcare costs and counterpoising the consequences of the medical resources shortage. Moreover, the continuous remote monitoring of PD patients with wearable or non-wearable sensors could potentially provide much more useful information and shed light on PD aspects that otherwise may not be perceived through follow-up appointments. To achieve that, more sensors could be deployed in larger cohorts to feed novel ML and DL models with data and achieve more accurate predictions and estimations in currently ill-addressed PD-related problems, overcoming the respective challenges. In this way, AI and IoT interventions may finally support precise medicine and help clinicians to propose personalized treatment schemes and potentially maximize patients’ responses to them.

## Data Availability

Not applicable.

## Abbreviations

The following abbreviations are used in this manuscript:

2D	Two Dimensions
3D	Three Dimensions
AdaBoost	Adaptive Boosting
ACC	Accuracy
ADL	Activity of Daily Living
AE	Autoencoder
AI	Artificial Intelligence
ALS	Amyotrophic Lateral Sclerosis
ANFIS	Adaptive Neuro-Fuzzy Inference System
ANN	Artificial neural Network
ANOVA	Analysis of Variance
AR	Autoregressive
ARMA	Autoregressive Moving Average
AU	Action Unit
AUC	Area Under the Curve
AUPR	Area under the Precision-Recall Curve
AUROC	Area Under the Receiver-Operating Characteristic Curve
Avg	Average
BDI	Beck Depression Inventory
BE	Backward Elimination
CAD	Computer-Aided Diagnosis
CART	Classification and Regression Tree
CFS	Correlation-based Feature Selection
CNN	Convolutional Neural Network
CT	Computed Tomography
CWT	Continuous Wavelet Transform
DA	Discriminant Analysis
DBN	Deep Belief Network
DCALSTM	Dual-modal Convolutional Neural Network + Attention Enhanced Long Short-Term Memory
DCNN	Dual-modal Convolutional Neural Network
DD	Other Movement Disorder
DESN	Deep Echo State Network
DFT	Discrete Fourier Transform
DL	Deep Learning
DNN	Deep Neural Network
DT	Decision Tree
DTW	Dynamic Time Warping
DWT	Discrete Wavelet Transform
ECG	Electrocardiography
EEG	Electroencephalography
EER	Equal Error Rate
ELM	Extreme Learning Machine
EMG	Electromyography
ESN	Echo State Network
ESS	Epworth Sleepiness Scale
ET	Essential Tremor
EWPT	Empirical Wavelet Packet Transform
EWT	Empirical Wavelet Transform
FA	Factor Analysis
FFT	Fast Fourier Transform
FIR	Finite Impulse Response
FLDA	Fisher Linear Discriminant Analysis
FNR	False Negative Rate
FoG	Freezing of Gait
FPR	False Positive Rate
FRP	Fuzzy Recurrent Plot
GBM	Gradient Boosting Machine
GD	Gradient Descent
GDM	Gradient Descent with Momentum
GM	Geometric Mean
GMM	Gaussian Mixture Model
GP	Gaussian Process
GRF	Ground Reaction Force
GRU	Gated Recurrent Unit
GS	Graph Sequence
H&Y	Hoehn and Yahr
HBNN	Hierarchical Bayesian Neural Network
HD	Huntington’s Disease
hHMM	Hierarchical Hidden Markov Model
HMCI	Healthy People with Mild Cognitive Impairment
HMM	Hidden Markov Model
HOA	Healthy Older Adult
HOG	Histogram of Oriented Gradients
IBk	Instance-Based with parameter k
ICC	Intraclass Correlation Coefficient
IIR	Infinite Impulse Response
IMU	Inertial Measurement Unit
IoT	Internet of Things
IPD	Idiopathic Parkinson’s Disease
JMIM	Joint Mutual Information Maximization
KELM	Kernel Extreme Learning Machine
KFD	Kernel Fisher Discriminant Analysis
kNN	k-Nearest Neighbors
LASSO	Least Absolute Shrinkage and Selection Operator
LDA	Linear Discriminant Analysis
LID	Levodopa-Induced Dyskinesia
LOSO	Leave One Subject Out
LR	Logistic Regression
LSTM	Long Short-Term Memory
MAE	Mean Absolute Error
MCC	Matthew’s Correlation Coefficient
MDS-UPDRS	Movement Disorder Society-Sponsored Revision of the Unified Parkinson’s Disease Rating Scale
MFCC	Mel-Frequency Cepstral Coefficient
ML	Machine Learning
MLP	Multilayer Perceptron
MMG	Mechanomyography
MML	Multi-source Multi-task Learning
MMTFL	Multiplicative Multi-Task Feature Learning
MoCA	Montreal Cognitive Assessment
MRI	Magnetic Resonance Imaging
mRMR	Maximum Relevance Minimum Redundancy
MS Kinect	Microsoft Kinect
mUPDRS	Motor subscale of the Unified Parkinson’s Disease Rating Scale
NB	Naïve Bayes
NN	Neural Network
OPF	Optimum Path Forest
PCA	Principal Component Analysis
PD	Parkinson’s Disease
PDD	Parkinson’s Disease Patients with Dementia
PDMCI	Parkinson’s Disease Patients with Mild Cognitive Impairment
PDNC	Parkinson’s Disease Patients with Normal Cognition
PET	Positron Emission Tomography
PKG	Personal KinetiGraph
PNN	Probabilistic Neural Network
PPV	Positive Predictive Value
PREC	Precision
PRISMA	Preferred Reporting Items for Systematic Reviews and Meta-Analyses
PSD	Power Spectral Density
PSG	Polysomnography
PSP	Progressive Supranuclear Palsy
Q-BTDNN	Q-Backpropagation for a Time Delay Neural Network
QDA	Quadratic Discriminant Analysis
RBD	Rapid Eye Movement Sleep Behavior Disorder
RBF	Radial Basis Function
REC	Recall
Res-Net	Residual Neural Network
RF	Random Forest
RGB	Red Green Blue
RGB-D	Red Green Blue-Depth
RMSE	Root Mean Square Error
RNN	Recurrent Neural Network
RUSBoost	Random UnderSampling Boosting
SBS	Sequential Backward Selection
SCG	Scaled Conjugate Gradient
SDE	Sparse Difference Embedding
SDH	Sum and Difference Histogram
sEMG	Surface-Electromyography
SENS	Sensitivity
SF-36	36-Item Short Form Survey
SFS	Sequential Forward Selection
SOM	Self-Organizing Map
SPDDS	Self-Assessment Parkinson’s Disease Disability Scale
SPEC	Specificity
STFT	Short-Time Fourier Transform
STL	Single Task Learning
SVM	Support Vector Machine
SVR	Support Vector Regression
TRIS	Treatment Response Index
TUG	Timed Up and Go
UDysRS	Unified Dyskinesia Rating Scale
UPDRS	Unified Parkinson’s Disease Rating Scale
VAE	Variational Autoencoder
VaP	Vascular Parkinsonism
VAS	Visual Analog Scale
XGBoost/XGB	Extreme Gradient Boosting

## Appendix A

**Table A1:** Studies based on inertial sensors.

Study	Problem	Dataset Population	Input Data	Analysis/Algorithms	Evaluation
Rastegari et al. (2020) [49]	Diagnosis	10 PD patients and 10 healthy controls from another study [166]	Accelerometer and gyroscope data from both ankles using SHIMMER sensors	Segmentation + bag-of-words features extraction based on sub-sequences clustering with k-medoid + SVM/DT/RF/kNN	Best performing: DT with ACC = 90%, PREC = 90%, REC = 90%
Zhang et al. (2020) [54]	Diagnosis	656 PD patients and 2148 healthy controls from the mPower study [126]	Gait features from smartphone sensors located at the pocket	Deep CNN	AUROC = 0.87
Juutinen et al. (2021) [50]	Diagnosis	29 PD patients and 29 healthy controls	Accelerometer and gyroscope signals from a waist-mounted smartphone while performing walking tests	Interpolation + low-pass filtering + smoothening + segmentation to strides + feature extraction + feature selection with mRMR/SFS/SBS + classification of strides with DT/Gaussian kernel/LDA/ensemble/kNN/LR/NB/SVM/RF + majority voting for subject classification	Best performing: SFS + kNN with ACC = 84.5%, SENS = 88.5%, SPEC = 81.3%
Fernandes et al. (2018) [51]	Parkinsonism diagnosis and differential diagnosis between idiopathic PD (IPD) and vascular parkinsonism (VaP)	15 IPD, 15 VaP patients and 15 healthy controls	Gait signals from wearable Physilog motion sensors placed on both feet and MOCA scores	Normalization + feature selection based on Kruskal–Wallis and Mann–Whitney tests with Bonferroni correction + features ranking with RFs + MLP/DBN	Best performing for parkinsonism detection: MLP with ACC = 99.33%, SENS = 93.33%; for IPD-VaP differential diagnosis: DBN with ACC = 73.33%, SENS = 73.33%, SPEC = 73.33%
Cuzzolin et al. (2017) [52]	Diagnosis and severity estimation in the SF-36 (0–100) scale	156 PD patients and 424 healthy controls	Signals from an IMU attached to the lower spine while walking	HMM + kNN	For diagnosis: ACC = 85.51%, F1-SCORE = 81.54%; for severity estimation: MAE = 27.81
Borzì et al. (2020) [70]	Severity estimation (MDS-UPDRS related to leg agility scores classification)	93 PD patients	Acceleration, angular velocity and orientation data from a smartphone application	Low-pass Butterworth filter + FFT + feature selection + DT/kNN/SVM/ANN	Best performing: ANN with ACC = 77.7%, AUROC = 0.92, r = 0.92, RMSE = 0.42, ICC = 0.82
Hssayeni et al. (2018) [96]	Fluctuation identification (on/off states detection)	12 PD patients (1st dataset) and 7 PD patients (2nd dataset)	Signals from an ankle-mounted triaxial gyroscope	FIR bandpass filter + LSTM	ACC = 73–77%, SENS = 63–75%, SPEC = 78–83%
Aich et al. (2020) [97]	Fluctuation identification (on/off states detection)	20 PD patients	Statistical and gait parameter features from 2 knee-worn accelerometers	Feature selection + RF/SVM/kNN/NB	Best performing: RF with ACC = 96.72%, PREC = 96.92%, REC = 97.35%, F1-SCORE = 0.97, AUROC = 0.99
Abujrida et al. (2020) [53]	Diagnosis and severity estimation regarding walking balance (MDS-UPDRS-2.12), shaking/tremor (MDS-UPDRS-2.10) and FoG	152 PD patients and 304 healthy controls from the mPower study [126]	Signals from smartphone embedded accelerometers and gyroscopes, demographics and lifestyle data	Segmentation + smoothening + feature extraction in time, frequency (FFT + PSD), wavelet (DWT) domain + RF/bagged trees/boosted tress/fine tree/cubic SVM/weighted kNN/LR/LDA	Best performing for diagnosis: RF with ACC = 95%, PREC = 94%, AUROC = 0.99; for walking balance: RF with ACC = 93%, PREC = 92%, AUROC = 0.97; for tremor: bagged trees with ACC = 95%, PREC = 95%, AUROC = 0.92; for FoG: bagged trees with ACC = 98%, PREC = 96%, AUROC = 0.98
Kim et al. (2015) [84]	FoG detection	15 PD patients	Gyroscopic and accelerometer data from smartphones placed at the waist, pocket and ankle	AdaBoost	SENS = 81.1–86%, SPEC = 91.5–92.5%
Ashour et al. (2020) [87]	FoG detection	10 PD patients	Accelerometer data from sensors placed on the ankles, knees and hips	Patient-dependent LSTM/{DWT/FFT + Patient-dependent SVM/ANN}	Best performing: LSTM with ACC = 68.44–98.89%
Torvi et al. (2016) [89]	FoG prediction in 1 s, 3 s and 5 s horizons	10 PD patients from Daphnet dataset	Accelerometer data from sensors placed on the ankles, the thighs and the trunk	LSTM/RNN with or without transfer learning	Best performing: LSTM + transfer learning with ACC = 85–95%
Arami et al. (2019) [90]	FoG prediction (2-class classification FoG/no-FoG and 3-class classification pre-FoG/FoG/no-FoG)	10 PD patients from Daphnet dataset	Accelerometer data from sensors placed on the ankles, the thighs and the trunk	Windowing + filtering + feature extraction + feature selection with mRMR/BE + features time series prediction with AR/ARMA + RBF-SVM/PNN	For 2-class classification: ACC = 94%, SENS = 93%, SPEC = 87%; for 3-class classification: ACC = 77%
Kleanthous et al. (2020) [91]	FoG detection (FoG/walking/transition from walking to FoG classification)	10 PD patients from Daphnet dataset	Accelerometer data from sensors placed on the ankles, the thighs and the trunk	Low pass Butterworth filter + Boruta algorithm + GBM + XGB for feature selection + XGB/RF/GBM/RBF-SVM/MLP	Best performing: RBF-SVM with ACC = 79.85%, SENS = 72.34–91.49%, SPEC = 87.36–93.62%
Li et al. (2020) [88]	FoG detection	10 PD patients from Daphnet dataset	Accelerometer data from sensors placed on the ankles, the thighs and the trunk	Filtering + segmentation + data augmentation + CNN with squeeze-and-excitation blocks for feature extraction + attention-enhanced LSTM	ACC = 98.1–99.7%, SENS = 95.1–99.1%, SPEC = 98.8–99.8% for generalized and personalized models with 10-fold cross-validation; AUC = 0.945, ACC = 91.9%, EER = 10.6% with LOSO validation
Halder et al. (2021) [92]	FoG states classification (pre-FoG, FoG, pre-post-FoG, no-FoG)	10 PD patients from Daphnet dataset	Accelerometer data from sensors placed on the ankles, the thighs and the trunk	Second-order Butterworth low-pass filtering + PCA + kNN/MLP/SVM	Best performing: kNN with ACC = 98.92%, SENS = 94.97%, SPEC = 99.19%, F1-SCORE = 95.25%, PREC = 95.55%
Palmerini et al. (2017) [93]	Pre-FoG detection (classification between gait and pre-FoG)	11 PD patients	Accelerometer and gyroscope signals from sensors placed at the lower-back and at the ankles	Windowing + feature extraction + LDA	SENS = 83%, SPEC = 67%, AUROC = 0.76
Borzì et al. (2021) [94]	FoG and pre-FoG detection	11 PD patients on and off therapy	A single angular velocity signal from 2 shins-mounted inertial sensors while performing TUG tests	Normalization + segmentation + wrapper feature selection + SVM/kNN/LDA/LR	Best performing: SVM with ACC = 85.5–86.1%, SENS = 84.1–85.5%, SPEC = 85.9–86.3%, F-SCORE = 73.4–74.6%
Shi et al. (2020) [85]	FoG detection	63 PD patients	Accelerometer, gyroscope and magnetometer signals from IMUs placed on both ankles and the spine while performing TUG tests	Time-series segmentation with overlapping windows + Morlet CWT/FFT/raw data + 1D-CNN/2D-CNN/LSTM	Best performing: CWT + 2D-CNN with ACC = 89.2%, SENS = 82.1%, SPEC = 96%, GM = 88.8%
Camps et al. (2018) [86]	FoG detection	21 PD patients	Signals from a waist-mounted IMU with accelerometer, gyroscope and magnetometer while performing several walking tests and ADLs	Spectral window stacking (with FFT) + RUSBoost/SVM-RBF/CNN	Best performing: CNN with ACC = 89%, SENS = 91.9%, SPEC = 89.5% and geometrical mean of SENS-SPEC = 90.6%
Ghassemi et al. (2018) [95]	Gait segmentation (strides detection)	10 PD patients for the 1st experiment and 34 PD patients for the 2nd experiment	Acceleration and angular velocity signals from foot-worn IMUs while walking in straight line (1st experiment) and walking, turning or performing other movements (2nd experiment)	Peak detection algorithm/Euclidean DTW/Probabilistic DTW/hierarchical HMM	Best performing for the 1st experiment: all with F-SCORE = 99.8–100%; For the 2nd experiment: hHMM with PREC = 98.5%, REC = 93.5%, F-SCORE = 95.9%
Kostikis et al. (2015) [55]	Diagnosis	25 PD patients and 20 healthy controls	Tremor measurements from accelerometer and gyroscope smartphone sensors	NB/LR/SVM/AdaBoost/C4.5/RF	Best performing: RF with SENS = 82%, SPEC = 90%, AUROC = 0.94
Williamson et al. (2021) [59]	Diagnosis	202 PD patients and 178 healthy controls from the UK Biobank dataset (https://www.ukbiobank.ac.uk/, accessed on 17 February 2022)	Acceleration signals from a wrist-worn sensor	Segmentation + automatic segments labelling (gait or low movement) + segmentation into frames + features extraction + GMM	SENS = 65–75%, AUROC = 0.85
Park et al. (2021) [60]	Diagnosis	25 PD patients and 21 healthy controls	Signals from IMUs attached to the thumb and index fingers while performing finger tapping, hand movements and rapid altering movements	Linear regression + correlation between motor parameters and UPDRS scores + DNN/LR for PD diagnosis	For motor parameters-UPDRS scores correlation: r = 0.838–0.849; Best performing for diagnosis: DNN with AUROC = 0.888–0.950
Talitckii et al. (2021) [65]	Differential diagnosis between PD and other extrapyramidal disorders	41 PD patients and 15 patients with other extrapyramidal disorders	Accelerometer, gyroscope and magnetometer signals form a dorsal-mounted sensor while performing UPDRS-related tasks	STFT + feature extraction + linear-PCA/RBF-PCA/poly-PCA/LDA/FA + RF/SVM/LR/LightGBM/stacked ensemble model	Best performing: standard classifier with ACC = 72–85%, PREC = 72–85%, REC = 77–100%, F1-SCORE = 76–88%
Varghese et al. (2020) [66]	PD or other movement disorders (DD) patients and healthy controls classification; PD patients and DD patients or healthy controls classification	192 PD patients, 75 DD patients and 51 healthy controls	Accelerometer data from 2 smartwatches placed at both hands and answers from electronic questionnaires distributed via smartphones	FFT + PCA + RBF-SVM/RF/ANN	Best performing for PD/DD detection: ANN with ACC = 89%, PREC = 94%, REC = 92%, F1-SCORE = 93%; for PD detection: RBF-SVM with ACC = 79%, PREC = 81%, REC = 89%, F1-SCORE = 85%
Loaiza Duque et al. (2020) [67]	Healthy and trembling subjects classification; PD-ET differential diagnosis	19 PD patients, 20 ET patients and 12 healthy controls	Angular velocity signals from smartphone built-in triaxial gyroscope with the help of a smartphone application	Kinematic features extraction + feature selection based on chi-square and unbiased tree + linear-SVM/LR/DA/NB/DT/ensemble subspace kNN	Best performing for trembling patients detection: ensemble subspace kNN with ACC = 97.2%, SENS = 98.5%, SPEC = 93.3%; for PD-ET differential diagnosis: linear-SVM with ACC = 77.8%, SENS = 75.7%, SPEC = 80%
Channa et al. (2021) [83]	Classification between PD patients with tremor and PD patients with bradykinesia and healthy controls	10 PD patients with tremor, 10 PD patients with bradykinesia and 20 healthy controls	Accelerometer and gyroscope signals from a smart bracelet	Butterworth bandpass IIR filter + FFT to extract both time and frequency domain features + feature selection based on ANOVA test + NN-SOMs clustering/kNN	Best performing: kNN with ACC = 91.7%, SENS = 83–100%, SPEC = 89–100%
Li et al. (2019) [57]	Diagnosis and severity (H&Y scores) estimation	13 PD patients and 12 healthy controls	Acceleration and angular velocity signals from a prototype handle for spoons with embedded inertial sensors	IIR filtering + windowing + feature extraction + normalization + kNN/Adaboost/RF/linear-SVM for diagnosis; RF regression for severity estimation	Best performing for diagnosis: linear-SVM with ACC = 92%, SENS = 92.31%, SPEC = 91.67%, AUROC = 0.98; for severity estimation: MAE = 0.166, r = 0.97
Koçer et al. (2016) [56]	Diagnosis and severity (H&Y scores) estimation	35 PD patients and 20 healthy controls	Resting tremor acceleration signals from the Nintendo Wii Remote (Wiimote)	Windowing + feature extraction (with FFT) + SVM	For diagnosis: ACC = 89%, PREC = 91%, REC = 94%; for severity estimation: ACC = 33–77%
Bazgir et al. (2015) [71]	Severity estimation (UPDRS scores classification)	52 PD patients	Accelerometer and gyroscope data from a smartphone placed at the wrist	Filtering + STFT + MLP trained with the back propagation algorithm	ACC = 91%, SENS = 89.6%, SPEC = 90.64%
Kim et al. (2018) [72]	Severity estimation (UPDRS scores classification)	92 PD patients	Accelerometer and gyroscope signals from a wrist-worn device	High pass filter + FFT + RF/NB/linear regression/DT/MLP/SVM/CNN	Best performing: CNN with ACC = 85%, kappa = 0.85, r = 0.93, RMSE = 0.35
Dai et al. (2021) [73]	Severity estimation (MDS-UPDRS scores classification)	42 PD patients and 30 healthy controls	Accelerometer, gyroscope and geomagnetic data from a finger-mounted sensor while measuring rest and postural tremor and during finger tapping	Denoising with an IIR bandpass filter + FFT + SVM/RF/kNN	Best performing: SVM with ACC = 96–97.33%, SENS = 96.36–100%, SPEC = 95–96.67%
Khodakarami et al. (2019) [74]	Severity estimation (UPDRS scores classification) and prediction of response to levodopa (absolute value and percentage)	151 PD patients and 174 healthy controls	Signals from the wrist-worn smartwatch of the Parkinson’s Kinectigraph system	Feature extraction + JMIM-based feature selection + (+PCA) LR/RBF-SVM/gradient boosting DTs	Best performing for severity estimation: LR with AUROC = 0.79–0.88, AUPR = 0.65–0.88; for absolute levodopa response estimation AUROC = 0.92 and AUPR = 0.87; for levodopa response percentage estimation AUROC = 0.82, AUPR = 0.73
Javed et al. (2018) [58]	Diagnosis and treatment response index (TRIS) estimation	19 PD patients and 22 healthy controls	Accelerometer and gyroscope data from both wrists using SHIMMER3 sensors while performing hand rotation tests before and after the dose administration	PCA/stepwise regression/LASSO regression + SVM/linear regression/DT/RF	Best performing for diagnosis: stepwise regression + SVM with ACC = 89%; for TRIS estimation: RMSE = 0.69, r = 0.84
Watts et al. (2021) [100]	PD patients classification according to their levodopa regimens and response	26 PD patients	Bradykinesia and dyskinesia-related signals from Personal KinetiGraph (PKG), demographics and MDS-UPDRS-III scores	k-means for clustering based on regimen features + RF for classification based on PKG features, demographics and MDS-UPDRS-III scores	ACC = 86.9%, SENS = 86.5%, SPEC = 87.7%, PPV = 95.3%, F1-SCORE = 90.7%, AUROC = 0.871
Pfister et al. (2020) [98]	On/Off/Dyskinesia motor states classification	30 PD patients	Data from a wrist-worn accelerometer in a free-living environment	Data augmentation + CNN/SVM/kNN/RF/MLP	Best performing: CNN with ACC = 65.4%, Kohen’s Kappa = 0.47, SENS = 64.45–66.68%, SPEC = 66.72–89.48%, F1-SCORE = 62.4–69.01%, 1vsALL ACC = 66.7–82.56%
Eskofier et al. (2016) [79]	Bradykinesia detection	10 PD patients	Accelerometer data from IMUs mounted on both hands	Timeseries segmentation + AdaBoost/PART/kNN/SVM/CNN-DNN	Best performing: DNN ACC = 90.9%
Shawen et al. (2020) [80]	Tremor and bradykinesia detection; severity estimation (UPDRS scores classification)	13 PD patients	Accelerometer and gyroscope signals from a flexible skin-mounted sensors and accelerometer signals from a wrist-worn smartwatch	Cubic spline interpolation + segmentation + high-pass filtering + time, frequency, entropy, correlation and derivative-based feature extraction + RF	For tremor detection: AUROC = 0.68–0.79; for bradykinesia detection: AUROC = 0.61–0.69; for tremor severity estimation: AUROC = 0.67–0.77; for bradykinesia severity estimation: AUROC = 0.59–0.66
San-Segundo et al. (2020) [81]	Tremor detection; tremor duration estimation	12 PD patients for laboratory set and 6 PD patients for in-the-wild set	Accelerometer signals from wrist-worn sensors	Downsampling + FFT + unsupervised non-negative tremor factorization + feature extraction manually/with a CNN + RF/MLP	Best performing for tremor detection: CNN + MLP with AUC = 0.887; for tremor duration estimation: MAE = 4.1–9.1%
Ibrahim et al. (2020) [82]	Tremor onset detection	13 PD patients	Signals from hand-mounted IMUs while performing 6 different rest, postural and motor tasks	Butterworth filtering + zero-phase shifting + Hilbert-Huang Transform + MLP	ACC = 92.9%, PREC = 98.7%, REC = 86.7%, SPEC = 98.9%, F1-SCORE = 0.923
Som et al. (2020) [61]	Diagnosis	152 healthy subjects for pre-training and 18 PD patients and 16 healthy controls for the final classification	Accelerometers signals from a wrist-worn sensor while performing various ADL for the pre-training + accelerometer signals from 6 sensors located at the sternum, the lumbar, both ankles and wrists while walking	Zero-centering + normalization + segmentation + feature extraction with pre-trained convolutional AE + PCA/global-average-pool layer + SVM/MLP	Best performing: MLP with ACC = 68.64–73.81%, PREC = 69.27–76.53%, REC = 68.64–73.81%, F1-SCORE = 67.65–73.89%
Ricci et al. (2020) [62]	Diagnosis	30 newly diagnosed untreated PD patients and 30 healthy controls	Acceleration, angular velocity and orientation signals from a network of 14 IMUs distributed in the whole body	Feature selection with ReliefF ranking and Kruskal–Wallis + NB/kNN/SVM	Best performing: SVM with ACC = 95%, PREC = 95.1%, AUROC = 0.95
De Vos et al. (2020) [69]	PD and PSP differential diagnosis	20 PD patients and 21 PSP patients	Accelerometer, gyroscope and magnetometer signals from 6 IMUs placed on the lumbar spine, the sternum, both wrists and feet	ANOVA + LASSO + LR/RF	Best performing: RF with ACC = 88%, SENS = 86%, SPEC = 90%
Moon et al. (2020) [68]	PD and ET differential diagnosis	524 PD patients and 34 ET patients	Balance and gait characteristics from 6 IMUs placed on the lumbar spine, the sternum, both wrists and feet	NN/SVM/kNN/DT/RF/LR	Best performing: NN with ACC = 89%, PREC = 61%, REC = 61%, F1-SCORE = 61%
Kuhner et al. (2017) [63]	Diagnosis; correlation with severity metrics	14 PD patients and 26 healthy controls	Fusion of accelerometer, gyroscope and magnetometer signals from XSens motion capture suit while performing several motor tests	RF with probability distributions for classification PCA for correlation	For diagnosis: ACC = 86–94.6%, SENS up to 91.5% and SPEC up to 97.2%; for correlation between the 1st pc and the UPDRS scores: r = 0.79
Caramia et al. (2018) [64]	Diagnosis and severity estimation (H&Y scores classification)	25 PD patients and 25 healthy controls	Accelerometer, gyroscope and magnetometer signals from 8 IMUs attached to both feet dorsum, thighs, shanks and to the chest and lumbar	Extraction of range of motion parameters/spatio-temporal parameters (+PCA) + LDA/NB/kNN/linear-SVM/RBF-SVM/DT + majority voting with equal weights/weights analogue to the individual accuracies	Best performing for diagnosis: majority voting with weights analogue to the individual accuracies with ACC = 96%; for severity estimation: RBF-SVM with ACC = 87.75–94.5%
Hssayeni et al. (2021) [76]	Severity (UPDRS-III scores) estimation	24 PD patients	Angular velocity from one wrist-mounted and one ankle-mounted inertial sensor	Gradient tree boosting/dual-channel LSTM with hand-crafted features and with or without transfer learning/1D-CNN-LSTM for raw signals/2D-CNN-LSTM for time-frequency data + ensemble learning	Best performing: ensemble of dual-channel LSTM with hand-crafted features and transfer learning, 1D-CNN-LSTM for raw signals and 2D-CNN-LSTM for time-frequency data with r = 0.79, MAE = 5.95
Butt et al. (2020) [77]	Severity (MDS-UPDRS III scores) estimation	64 PD patients and 50 healthy controls	Gyroscope and geomagnetic data from 4 wrist-mounted IMUs and 1 foot-mounted IMU	Kolmogorov–Smirnov test + Mann–Whitney U-test + normalization + CFS/PCA ranker/correlation attribute evaluation/chi-square attribute evaluation/wrapper subset evaluation + SVR/RF/LR/ANFIS	Best performing: CFS + ANFIS with r = 0.814, RMSE = 0.101
Mirelman et al. (2021) [75]	Severity estimation (H&Y stages classification)	332 PD patients and 100 healthy controls	Accelerometer and gyroscope signals from sensors placed on the ankles, the wrists and the lower back while performing various walking tests + demographic data	Low-pass Butterworth filter + feature selection based on RF permutation importance/neighborhood component analysis/mRMR + RUSBoost + DT/QDA for weak learner	SENS = 72–84%, SPEC = 69–80%, AUROC = 0.76–0.90
Stamate et al. (2018) [78]	Identification of failures to follow the UPDRS-III movement protocol	12 PD patients	Motor signals from smartphone sensors with the cloudUPDRS application	Filtering + frequency transformations + extra tree/Bernoulli NB/Gaussian NB/MLP/RF/Gradient Boosting/Bagging/AdaBoost/RCNN	ACC = 78%, F1-SCORE = 82%, AUROC = 0.87
Belgiovine et al. (2018) [99]	L-dopa induced dyskinesia detection	18 PD patients	Accelerometer and angular velocity data from smartphone placed on the wrist (for upper-limb experiment) or on the hip (for lower-limb experiment)	z-score normalization + DT/Gaussian-SVM/linear-SVM	Best performing: SVM (with both kernels) with ACC = 65.0–82.0%, MACRO F1-SCORE = 0.65–0.82

**Table A2:** Studies based on pressure and force sensors.

Study	Problem	Dataset Population	Input Data	Analysis/Algorithms	Evaluation
Aversano et al. (2020) [102]	Diagnosis and severity estimation (H&Y scores classification)	93 PD patients and 73 healthy controls from 3 merged datasets [106]	Vertical GRF signals from 8 sensors located underneath each foot	Feed-forward DNN	For diagnosis: ACC = 99.29–99.52%; for severity estimation: ACC = 98.57–99.1%
El Maachi et al. (2020) [103]	Diagnosis and severity estimation (H&Y scores classification)	93 PD patients and 73 healthy controls from 3 merged datasets [106]	Vertical GRF signals from 8 sensors located underneath each foot	Gait segmentation + 1D-CNN/DNN/MLP/NB/RF	Best performing for diagnosis: 1D-CNN with ACC = 98.7%, SENS = 98.1%, SPEC = 100%; for severity estimation: ACC = 85.3%, PREC = 87.3%, REC = 85.3%, F1-SCORE = 85.3%
Xia et al. (2020) [104]	Diagnosis and severity estimation (H&Y scores classification)	93 PD patients and 73 healthy controls from 3 merged datasets [106]	Vertical GRF signals from 8 sensors located underneath each foot	Dual-modal CNN + attention-enhanced LSTM (DCALSTM)/baseline DL models removing one of the previous stages (DCNN/DALSTM/DCLSTM/CNN-LSTM)/feature-based models	Best performing for diagnosis: DCALSTM with ACC = 99.07–99.31%, SENS = 99.10–99.35%, SPEC = 98.98–99.35%; for severity estimation: ACC = 98.03–99.01%
Nancy Jane et al. (2016) [101]	Severity estimation (H&Y scores classification)	93 PD patients and 73 healthy controls from 3 merged datasets [106]	Vertical GRF signals from 8 sensors located underneath each foot	Q-backpropagation/Levenberg–Marquardt/GD/GDM/SCG for a time delay NN	Best performing: Q-BTDNN with ACC = 90.91–92.19%
Balaji et al. (2021) [105]	Severity estimation (H&Y scores classification)	93 PD patients and 73 healthy controls from 3 merged datasets [106]	Vertical GRF signals from 8 sensors located underneath each foot	Normalization + spatiotemporal feature extraction + Shapiro–Wilk test + feature selection + kNN/NB/Bagging classifier/SVM	Best performing: SVM with ACC = 98.8%, SENS = 96.6%, SPEC = 99.6%, PPV = 99.1%, FPR = 3.4%, PREC = 99.1%, F-SCORE = 97.8%, MCC = 0.98
Papavasileiou et al. (2017) [107]	Differential diagnosis between PD, post-stroke patients and healthy controls	5 PD patients, 3 post-stroke patients and 3 healthy controls	Ground contact force data from barometric pressure sensors placed on both feet	Multiplicative multi-task feature learning (MMTFL)/single task learning (STL)	Best performing: MMTFL AUROC = 0.880–0.994
Khoury et al. (2019) [108]	Diagnosis and differential diagnosis between PD, HD, ALS and healthy controls	93 PD patients and 73 healthy controls from 3 merged datasets [106] for diagnosis; 15 PD patients, 20 HD patients, 13 ALS patients and 16 healthy controls for differential diagnosis	Vertical GRF signals from 8 sensors located underneath each foot	Feature selection with RF-based wrapper method + kNN/DT/RF/NB/SVM/k-means/GMM	Best performing for diagnosis: kNN/RF/SVM with ACC = 81.25–90.91%, PREC = 81.43–89.41%, REC = 71.48–88.35%, F-SCORE = 79.45–86.83%; for PD vs. ALL differential diagnosis: kNN with ACC = 90%, PREC = 90.18%, REC = 90%, F-SCORE = 90.09%

**Table A3:** Studies based on image and depth sensors.

Study	Problem	Dataset Population	Input Data	Analysis/Algorithms	Evaluation
Guayacán et al. (2020) [111]	Diagnosis	11 PD patients and 11 healthy controls	Video recordings while walking	3D spatio-temporal CNN	ACC = 88–90%
Reyes et al. (2019) [109]	Diagnosis	88 PD patients and 94 healthy controls	Gait samples from MS Kinect	Cropping noisy parts + LSTM/1D-CNN/CNN-LSTM	Best performing: CNN-LSTM with ACC = 83.1%, PREC = 83.5%, REC = 83.4%, F1-SCORE = 81%, Kappa = 64%
Buongiorno (2019) [110]	Diagnosis and severity estimation (mild vs. moderate)	16 PD patients and 14 healthy controls	Postural and kinematics features from MS Kinect v2 sensor while performing 3 motor exercises (gait, finger and foot tapping)	Feature selection + SVM/ANN	Best performing for diagnosis: gait-based ANN with ACC = 89.4%, SENS = 87.0%, SPEC = 91.8%; for severity estimation: ACC = 95.0%, SENS = 90.0%, SPEC = 99.0%
Grammatikopoulou et al. (2019) [117]	Severity estimation (UPDRS scores classification)	12 advanced PD patients and 6 PD patients in initial stage	Skeletal features from MS Kinect v2 RGB videos while playing an exergame	Transformation to local coordinate system + two parallel LSTMs (the 1st trained with raw joint coordinates and the 2nd with joint line distances)	ACC = 77.7%
Tucker et al. (2015) [122]	Medication adherence estimation (on/off medication classification)	7 PD patients	Skeletal joints 3D position, velocity and acceleration from MS Kinect	C4.5 DT for generalized model; C4.5 DT, RF, SVM, IBk for personalized models	for generalized model: ACC = 36.2–77.9%; for personalized models: ACC = 67.7–100%
Li et al. (2018) [120]	TUG subtasks segmentation and time estimation for each subtask	24 PD patients	Video recordings while performing TUG tests	Pose estimation with OpenPose/Iterator Error Feedback + SVM/LSTM	Best performing: OpenPose + LSTM with ACC = 93.1%, PREC = 80.8–97.5%, REC = 86.3–97%, F1-SCORE = 83.5–97.3% for subtasks segmentation and MAE = 0.32–1.07 for time estimation
Wei et al. (2019) [123]	Development of a virtual physical therapist: movement recognition (repetitions and sub-actions detection), patient’s errors identification (satisfactory/non-satisfactory performance), task recommendation (regress/repeat/progress)	35 PD patients	Motion data recorded by MS Kinect v2 sensor while performing 3 balance/agility tasks	HMM for repetitions and sub-actions detection + linear-SVM for movement errors identification + {majority undersampling/minority oversampling/decision threshold adjustment/hybrid oversampling with feature standardization and interpolation + RF} for task recommendation	For repetitions detection: ACC = 97.1–99.4%; for sub-actions segmentation: SENS = 88.4–96.9%, SPEC = 97.2–98.8%; for errors identification: ACC = 86.3–94.2%; best performing for tasks recommendation: hybrid oversampling + RF with ACC = 81.8–95.7%, FPR = 2.8–5.4%
Hu et al. (2019) [121]	FoG detection	45 PD patients	Videos collected while performing TUG tests	Graph representation of videos + pretrained features (Res-Net 50 vertex, C3D vertex and context features) + graph sequence-RNN (Bi-directional GS-GRU/Bi-directional GS-LSTM/forward GS-GRU/forward GS-LSTM) + fusion	Best performing: linear fusion of Bi-directional GS-GRU with context model with AUC = 0.90, SENS = 83.8%, SPEC = 82.3%, ACC = 82.5%, Youden’s J = 0.66, FPR = 17.7%, FNR = 16.2%
Li et al. (2018) [118]	Binary classification between pathological (PD/LID) and normal motion; multiclass classification (PD with LID, PD without LID and normal); levodopa-induced dyskinesia severity (UDysRS-III scores) estimation; parkinsonism severity (UPDRS-III scores) estimation	9 PD patients	2D-Video recordings while performing communication and drinking tasks (for dyskinesia detection) and while performing leg agility and toe tapping tasks (for parkinsonism detection)	Convolutional pose estimators + RF	For binary classification: AUC = 0.634–0.930, F1-SCORE = 50–90.6%; for multiclass classification: ACC = 71.4%, SENS = 83.5–96.2%, SPEC = 68.4–95.7%; for UDysRS-III estimation: RMSE = 2.906, r = 0.741; for UPDRS-III estimation: RMSE = 7.765, r = 0.53
Vivar-Estudillo et al. (2018) [112]	Diagnosis	18 PD patients and 22 healthy controls	Position, velocity and rotation data regarding hand movements from leap motion sensor	Texture features extraction with SDH + kNN/SVM/DT/LDA/LR/ensembles	Best performing: bagged tree with ACC = 98.62%, SENS = 98.43%, SPEC = 98.80%, PREC = 98.80%
Moshkova et al. (2020) [113]	Diagnosis	16 PD patients and 16 healthy controls	Signals from leap motion sensor while performing hand motor tasks according to the MDS-UPDRS-III	Features extraction + kNN/SVM/DT/RF	Best performing: SVM with ACC = 98.4% when features are extracted from all the tasks
Ali et al. (2020) [114]	Diagnosis; classification between PD patients with medication, without medication and healthy controls	87 PD patients with medication, 119 PD patients without medication and 139 healthy controls	Videos while performing hand motor tasks	Segmentation to frames + temporal segmentation with CNN + spatial segmentation with CNN-AE + FFT for feature extraction + SVM	Best performance when combining 2 tasks for diagnosis: ACC = 91.8%; for 3-class classification: ACC = 73.5%
Liu et al. (2019) [119]	Severity estimation (Bradykinesia-related MDS-UPDRS scores classification)	60 PD patients	Video recordings while performing hand motor tests	Pose estimator NN + feature extraction + kNN/RF/linear-SVM/RBF-SVM	Best performing: RBF-SVM with ACC = 89.7%, PREC = 20–100%, REC = 60–100%, F1-SCORE = 33.3–100%
Rajnoha et al. (2018) [116]	Diagnosis	50 PD patients and 50 age-matched healthy controls	Face images extracted from video recordings	HOG for face detection + CNN for embeddings generation + kNN/DT/RF/XGBoost/SVM	Best performing: DT:ACC = 67.33% with leave-one-out cross validation, RF: ACC = 60.7–85.92% with train-test split
Jin et al. (2020) [115]	Diagnosis	33 PD patients and 31 elderly healthy subjects	Short videos while imitating images of smiley people	Splitting videos to frames + coordinate points extraction with Face++ + transformation from absolute to relative coordinates + features extraction + LASSO + LR/SVM/DT/RF/LSTM/RNN	Best performing: SVM with PREC = 99%, REC = 99%, F1-SCORE = 99%

**Table A4:** Studies based on voice or audio sensors.

Study	Problem	Dataset Population	Input Data	Analysis/Algorithms	Evaluation
Zhang et al. (2017) [127]	Diagnosis	23 PD patients and 4 healthy controls from the Oxford’s dataset (https://archive.ics.uci.edu/ml/datasets/parkinsons+telemonitoring, accessed on 17 February 2022); 20 PD patients and 20 healthy controls from the Istanbul dataset (https://archive.ics.uci.edu/ml/datasets/Parkinson%27s+Disease+Classification, accessed on 17 February 2022)	Vocal measurements from a smartphone application	Stacked AEs + KELM/linear-SVM/MLP-SVM/RBF-SVM/kNN/NB/CART/LDA	Best performing: kNN with ACC = 94–98%
Zhang et al. (2018) [124]	Diagnosis	500 PD patients and 500 healthy controls from the mPower study [126]	Vocal measurements from a smartphone application	DT-STFT + LSTM/CNN	Best performing: CNN with ACC = 90.45%
Tougui et al. (2020) [125]	Diagnosis	453 PD patients and 1037 healthy controls from the mPower study [126]	Vocal measurements from a smartphone application	Time, frequency and cepstral domain (with DFT) features + feature selection with ANOVA/LASSO + linear-SVM/kNN/RF/XGBoost	Best performing: LASSO + XGBoost with ACC = 95.78%, SENS = 95.32%, SPEC = 96.23%, F1-SCORE = 95.74%
Almeida et al. (2019) [128]	Diagnosis	64 PD patients and 35 healthy controls	Audio recordings from acoustic cardioid and smartphone	Phonation/speech/unvoiced/voiced features + kNN/MLP/OPF/SVM	Best performing: kNN based on phonation features with ACC = 92.94–94.55%, SENS = 92.94–94.55%, SPEC = 89.21–94.26%, AUROC = 0.87–0.92
Arora et al. (2021) [129]	Differential diagnosis between PD patients, patients with RBD and healthy controls; severity (MDS-UPDRS, MoCA, ESS, BDI and VAS scores) estimation	335 PD patients, 112 patients with RBD and 92 healthy controls	Speech recording from smartphones	Segmentation + feature extraction + feature selection + RF	For all the pairwise classifications: SENS = 59.4–74.9%, SPEC = 67.4–73.2%; for severity estimation: MAE = 1–8 (MDS-UPDRS), MAE = 1–14 (MDS-UPDRS I-III), MAE = 1–2 (MoCA), MAE = 2–3 (ESS), MAE = 1–5 (BDI), MAE = 6.5–10 (VAS)
Bayestehtashk et al. (2015) [132]	Severity (mUPDRS 0–108 scores) estimation	168 PD patients	Speech recordings from a portable device	Feature extraction with harmonic model + Ridge/LASSO regression/linear-SVR	Best performing: ridge regression with MAE = 5.5, explained variance = 61%
Yoon et al. (2019) [130]	Severity (UPDRS 0–176 scores) estimation	42 PD patients	Phonation and speech recordings from an at-home testing device	Standardization + feature extraction + {one-model-fits-all approaches with DT/GP/linear regression/SVR/ensemble}/{single learning approaches with DT/GP/linear regression/SVR/ensemble}/positive transfer learning based on Bayesian parameter transfer model	Best performing: positive transfer learning with MAE = 2–3 approximately
Raza et al. (2021) [131]	Severity (UPDRS and mUPDRS scores) prediction in 6 months	42 PD patients	Phonation and speech recordings from an at-home testing device	Feature extraction + XGBoost	MAE = 6.45 (UPDRS), MAE = 5.09 (mUPDRS)

**Table A5:** Studies based on other types of sensors.

Study	Problem	Dataset Population	Input Data	Analysis/Algorithms	Evaluation
Rahman et al. (2020) [133]	Diagnosis	5 PD patients and 5 healthy controls	EEG signals from a portable headset with sensors placed at the forehead while watching 4 videos which provoke 4 different emotions	Feed-forward NN trained with Adam optimization algorithm	ACC = 96.5%, PREC = 95.5%, REC = 97%, F1-SCORE = 97.6%
Kleinholdermann et al. (2021) [134]	Severity (MDS-UPDRS III scores) estimation	45 PD patients	sEMG signals from a wrist-worn band while performing a simple tapping activity	Windowing + feature extraction + linear regression/poly-SVM/kNN/RF	Best performing: RF regression with r = 0.739
Capecci et al. (2019) [135]	Emotion (positive/negative) recognition	36 PD patients	Body temperature, heart rate and galvanic response from smartwatch sensors	Linear-SVM/poly-SVM/RBF-SVM	Best performing: RBF-SVM with ACC = 88.6–91.3%
Lacy et al. (2018) [136]	Diagnosis	49 PD patients and 41 healthy controls (1st dataset); 58 PD patients and 29 healthy controls (2nd dataset)	Position measures from 2 electromagnetic sensors located at the thumb and index finger while performing finger-tapping tests	Low-pass Butterworth filtering + velocity and acceleration features extraction from derivatives + ESN	AUROC = 0.802
Picardi et al. (2017) [137]	Diagnosis; classification between different cognition levels (PD patients with normal cognition-PDNC, PD patients with mild cognitive impairment-PDMCI and PD patients with dementia-PDD)	22 PDNC, 23 PDMCI, 10 PDD and 30 age-matched healthy controls	Flexion signals from a glove with finger-mounted sensors and position and orientation information from a wrist-worn tracking system	Feature extraction + Cartesian Genetic Programming/SVM/ANN	Similar performance for all the algorithms: AUROC = 0.72–0.99 for all the pair-wise classifications
Memedi et al. (2015) [138]	Symptom detection (bradykinesia/dyskinesia)	65 advanced PD patients	Spatiotemporal features from spiral drawings, produced with a touchscreen telemetry device	PCA + MLP/RF/RBF-SVM/linear-SVM/LR	Best performing: MLP with ACC = 84.0%, SENS = 75.7%, SPEC = 88.9%, AUROC = 0.86, weighted Kappa = 0.65
Pham et al. (2019) [139]	Diagnosis	42 PD patients and 43 healthy controls from newQWERTY MIT-CSXPD database (https://www.physionet.org/content/nqmitcsxpd/1.0.0/, accessed on 17 February 2022)	keystroke logs time series	CNN/LSTM/CNN-GoogleNet/CNN-AlexNet/LSTM-fuzzy recurrent plots (FRP)	Best performing: LSTM-FRP (m = 3) with ACC = 65.14–81.90%, SENS = 66.67–95%, SPEC = 63.33–66.67%
Matarazzo et al. (2019) [140]	Medication response detection (improved/not changed) and medication response prediction in 21 weeks	29 PD patients and 30 age-matched healthy controls	Keystroke logs with the help of neuroQWERTY software	RNN	For medication response detection: ACC = 76.5%, AUROC = 0.75, kappa = 0.47; for medication response prediction: AUROC = 0.69–0.75

**Table A6:** Studies based on the combination of previous types of sensors.

Study	Problem	Dataset Population	Input Data	Analysis/Algorithms	Evaluation
Aharonson et al. (2018) [141]	Diagnosis	22 PD patients and 20 healthy controls	Signals from sensors mounted on a support walker (2 encoders on the wheels, 2 force sensors underneath the hand grips and a tri-axial accelerometer) while performing two walking tests	Filtering + wavelet denoising for accelerometer signals + differentiation for encoder signals + ranked feature selection/PCA + k-means/FLDA	Best performing: PCA + FLDA with SENS = 91–96%, SPEC = 95–100%
Pardoel et al. (2021) [142]	FoG detection (pre-fog, transition, FoG, total-FoG, no-FoG events classification)	11 PD patients	Accelerometer and gyroscope signals from 4 foot-mounted sensors and plantar pressure distribution data from in-sole sensors	Data windowing + feature extraction in time and frequency domain with FFT and DWT + feature selection with mRMR/Relief-f + RUSBoost	For total-FoG/no-FoG classification: SENS = 61.9–78%, SPEC = 83.2–91.6%; for FoG/no-FoG classification: SENS = 81.4–98.5%, SPEC = 83.2–91.6%
Wu et al. (2020) [143]	Severity estimation (UPDRS-III scores classification)	17 PD patients	Acceleration signals from hand-mounted sensors and displacement signals from detection devices	Detrending + Wavelet transform + PCA + linear regression/SVM/NN/RF	Best performing: NN with ACC = 91.18–95.30%
Cole et al. (2014) [144]	Tremor and dyskinesia detection; severity estimation for tremor and dyskinesia	8 PD patients and 4 healthy controls	Acceleration and electromyography signals	Dynamical DNN/SVM/HMM for tremor and dyskinesia detection; Bayesian maximum likelihood classifier for severity estimation	Best performing for tremor detection: HMM with global error = 6.1%; for dyskinesia detection: DNN with global error = 8.8%; for tremor severity estimation: SENS = 95.2–97.2%, SPEC = 97.1–99.3%; for dyskinesia severity estimation: SENS = 91.9–95%, SPEC = 94.6–98.6%
Hossen et al. (2012) [145]	Differential diagnosis between PD and ET patients	39 PD patients and 41 ET patients	Signals from a hand-mounted accelerometer and 2 sEMG sensors placed at the forearm flexors and extensors	Filtering + feature extraction with SDE based on wavelet decomposition + MLP trained with the back-propagation algorithm	ACC = 91.6%, SENS = 95%, SPEC = 88.2%
Tahafchi et al. (2019) [146]	FoG detection	4 PD patients	Accelerometer and gyroscope signals from 2 foot-mounted IMUs and EMG signals from 2 Shimmer modules	Fully connected NN	AUC = 0.906–0.963
Huo et al. (2020) [147]	Diagnosis; Severity (UPDRS scores) estimation; PD patients with UPDRS > 0 and PD patients with UPDRS = 0 (due to DBS) classification	23 PD patients and 10 healthy controls	Bio-signals from hand-placed force sensor, 3 IMUs and 4 MMG sensors during different symptoms measurements (elbow rigidity, wrist rigidity, bradykinesia, kinetic tremor, postural tremor, rest tremor)	Voting classifier of 3 best performing basic classifiers (kNN, MLP and AdaBoost)	For severity estimation: ACC = 80.1–91.8% (avg = 85.4%); for diagnosis: ACC = 95.0–98.9% (avg = 96.6%); for PD patients with UPDRS > 0 and UPDRS = 0 classification: ACC = 85.2–91.1% (avg = 89.0%)
Yu et al. (2018) [148]	Severity estimation; Fall risk detection	22 PD patients	Signals from accelerometers, gyroscopes and thermometers embedded in 5 sensors attached to the chest, both thighs and feet while performing TUG tests	CNN for multi-source multi-task learning/single feature-based assessment + kNN/SVM/NB	Best performing for severity estimation: CNN for MML with RMSE = 0.060; for fall risk detection: PREC = 92.5%, REC = 95.8%, F-SCORE = 94%
Sajal et al. (2020) [149]	Diagnosis and severity estimation (voice and rest tremor MDS-UPDRS scores classification)	52 PD patients for tremor measurements; 23 PD patients and 8 healthy subjects for voice measurements (https://archive.ics.uci.edu/ml/datasets/parkinsons, accessed on 17 February 2022)	Rest tremor data from a smartphone built-in accelerometer and vowel phonation recordings from a smartphone	For tremor data: detrending + wavelet filtering For vocal data: bandpass filter + down-sampling + mRMR feature selection algorithm + kNN/SVM/NB + majority voting	Best performing for severity estimation: kNN with ACC = 93.7%, SENS = 94.6%, SPEC = 93.7% (vocal features), ACC = 90.5–98.5%, SENS = 87.5–94%, SPEC = 96–100% (tremor features); for diagnosis: kNN + SVM + NB ensemble averaging with ACC = 99.8%
Oung et al. (2018) [150]	Severity estimation (healthy/mild/moderate/severe classification)	15 healthy controls, 20 PD patients with mild severity, 20 PD patients with moderate severity, 15 PD patients with severe symptoms	Accelerometer, gyroscope and magnetometer data from 4 wrist- and limb-mounted IMUs and speech signals recorded with a headset	Segmentation + EWT for motor signals/EWPT for speech signals + Hilbert transformations + wavelet energy and entropy-based feature extraction + kNN/PNN/ELM	Best performing: ELM with ACC = 92.45–95.93%
Papadopoulos et al. (2020) [151]	Diagnosis; tremor/fine-motor impairment detection	14 PD patients and 8 healthy controls (1st dataset), 26 PD patients and 131 healthy controls (2nd dataset)	Accelerometer data from a smartphone sensor; typing dynamics from a smartphone virtual keyboard	Filtering + feature extraction with 1D-CNN for accelerometer data and fully connected NN for keystroke data + attention pooling module + NN classifiers	For tremor/fine-motor impairment detection on the 1st dataset: SENS = 85.4–92.8%, SPEC = 84.2–93.6%, PREC = 92.1–93%; for diagnosis on the 2nd dataset: ensemble of 10 models with AUROC = 0.834–0.868, SENS/SPEC = 60%/91.7–92%/68.9%
Heidarivincheh et al. (2021) [152]	Diagnosis	5 PD patients (on medication) and 5 healthy controls	Acceleration signals from a wrist-worn sensor and silhouette images from an RGB-D camera while cooking	{Convolutional VAEs + dense layers (MCPD-Net)}/CNN/unimodal VAE/RF/LSTM/other multimodal models	Best performing: MCPD-Net with PREC = 71%, REC = 77%, F1-SCORE = 66%
Wahid et al. (2015) [153]	Diagnosis	23 PD patients and 26 healthy controls	Spatial-temporal gait features captured by a video-camera and GRF from force platforms	Filtering + Normalization through multiple regression + KFD/NB/kNN/SVM/RF	Best performing: RF with AUROC = 0.96, ACC = 92.6%, SENS = 96%, SPEC = 90%
Albani et al. (2019) [154]	Diagnosis and severity estimation (UPDRS scores classification)	25 PD patients and 15 healthy controls for the final testing of the pre-trained models	Video recordings of upper-limbs from an RGB-D camera and accelerometer, gyroscope and magnetometer signals from 3 wearable sensors attached to the thighs and the chest	NB/LDA/MNR/kNN/poly-SVM for upper-limb classification (1st pretrained model); (PCA) + kNN/SVM for lower-limb classification (2nd pretrained model)	Best performing for diagnosis: SVM (1st model) + kNN (2nd model) with ACC = 91.5–98.6%; for severity estimation: ACC = 60.7–79.1%
Joshi et al. (2018) [155]	Facial expressivity estimation (classification and regression)	117 PD patients	Short interview audio-video clips	Audio features extraction (MFCC) + visual features extraction (AU statistics) + HBNN classification/regression + contextual information (gender/sentiment)	Best performing for classification: HBNN-sentiment with F1-SCORE = 0.55; for regression: MAE = 0.48
Barth et al. (2012) [159]	Diagnosis	18 PD patients and 17 healthy controls	Signals form a smart pen (acceleration, grip force, refill force, vibration sound); gyroscope and accelerometer data from a shoe-mounted IMU	Chebyshev low pass filter + linear forward feature selection with CFS/backtracking facility + LDA/linear-SVM/AdaBoost	Best performing: linear forward feature selection with CFS + AdaBoost with classification rate = 97%, SENS = 100%, SPEC = 94%
Xu et al. (2020) [156]	Diagnosis	31 PD patients and 35 healthy controls from an extended HandPD dataset (http://wwwp.fc.unesp.br/~papa/pub/datasets/Handpd/, accessed on 17 February 2022)	Pressure, tilt and acceleration signals from a smart pen while performing 6 different handwriting tasks	PCA + RF + voting scheme	ACC = 88.8–89.4%, SENS = 83.7–84.5%, SPEC = 93.4–93.7%, F1-SCORE = 87.2–87.7%
Gallicchio et al. (2018) [157]	Diagnosis	61 PD patients and 15 healthy controls from a UCI dataset (https://archive.ics.uci.edu/ml/datasets/Parkinson+Disease+Spiral+Drawings+Using+Digitized+Graphics+Tablet, accessed on 17 February 2022)	Pen position, pressure and grip angle while sketching spirals on a tablet	Deep ESN/shallow ESN + ensemble learning or not	Best performing: ensemble of DESNs with ACC = 89.33%, SENS = 90%, SPEC = 80%
Pereira et al. (2016) [158]	Diagnosis	14 PD patients and 21 healthy controls from an extended HandPD dataset (http://wwwp.fc.unesp.br/~papa/pub/datasets/Handpd/, accessed on 17 February 2022)	Various signals from a smart pen (microphone, finger grip, axial pressure of ink refill, tilt and acceleration) while drawing spirals and meanders	CNN architectures (ImageNet/CIFAR-10/LeNet)/OPF	Best performing for meander dataset: ImageNet with ACC = 84.74–87.14%; for spiral dataset: OPF with ACC = 77.92–83.77%
Schwab et al. (2019) [160]	Diagnosis	1853 subjects (PD patients and healthy subjects) from the mPower study [126]	Touchscreen data from a tapping activity, accelerometer data from a walking activity, performance in a memory game, vocal measurements and demographics from a mobile application	RF/CNN/RNN for each test + evidence aggregation model combining evidence from all the different tests	AUROC = 0.85, AUPR = 0.87, F1-SCORE = 82%
Prince et al. (2019) [161]	Diagnosis	1513 subjects (PD patients and healthy subjects)	Touchscreen data from a tapping activity, accelerometer data from a walking activity, performance in a memory game and vocal measurements from a mobile app	LR/RF/DNN/CNN for each test + ensemble learning combining the previous classifiers	ACC = 82.0%, F1-SCORE = 87.1%
Cook et al. (2015) [162]	Diagnosis; classification between healthy older adults and PD patients with and without mild cognitive impairment (HOA/PDNC/HMCI/PDMCI)	50 HOA and 25 PD patients for diagnosis; 18 HOA, 16 PDNC, 9 PDMCI, 9 HMCI for their classification	Signals from ambient sensors in a smart house environment (infrared motion sensors on the ceiling, light sensors, magnetic door sensors, temperature sensors and vibration sensors on selected items); accelerometer, gyroscope and magnetometer data from hand- and ankle- mounted sensors	No dimensionality reduction technique/PCA/k-means clustering/random resampling + DT/NB/RF/SVM/Ada-DT/Ada-RF	Best performing for diagnosis: k-means clustering + Ada-DT with ACC = 80%, AUC = 0.84; for the multiclass classification: random resampling + Ada-DT with ACC = 86%, AUC = 0.97
